# Co-assembly and self-sorting are not mutually exclusive in enantiomeric peptide systems

**DOI:** 10.1039/d6sc04454a

**Published:** 2026-08-27

**Authors:** Simona Bianco, Ravi R. Sonani, Libby J. Marshall, Alex S. Loch, James Doutch, Edward H. Egelman, Dave J. Adams

**Affiliations:** a MAX IV Laboratory, Lund University Lund 224 84 Sweden simona.bianco@maxiv.lu.se; b School of Chemistry, University of Glasgow Glasgow G12 8QQ UK dave.adams@glasgow.ac.uk; c Department of Biochemistry and Molecular Genetics, University of Virginia Charlottesville VA 22903 USA; d ISIS Pulsed Neutron and Muon Source Harwell Science and Innovation Campus Didcot OX11 0QX UK

## Abstract

Co-assembly and self-sorting in multicomponent systems are typically treated as mutually exclusive outcomes. Here, we show that this distinction is incomplete. Using enantiomeric peptide nanotubes, we demonstrate that a single co-assembled structure forms only at an equimolar composition, yet remains internally self-sorted into compositionally distinct domains. Contrast-matched neutron scattering directly reveals this segregation and shows that the co-assembled structures adopt layered architectures rather than simple molecular-level mixing. These results establish that co-assembly and self-sorting can coexist within a single supramolecular object across length scales, providing a general framework for understanding and controlling multicomponent self-assembly.

## Abstract 2

Contrast-matched neutron scattering shows that co-assembly and self-sorting are not mutually exclusive, revealing internally self-sorted domains within a single co-assembled peptide nanotube architecture.

## Graphical abstract

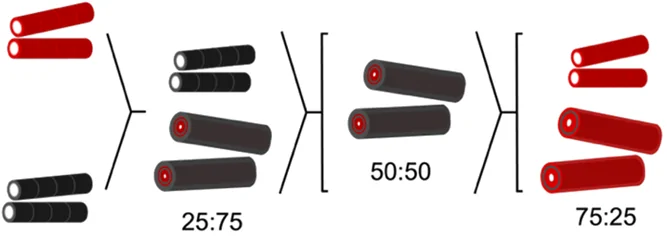

## Introduction

When two self-assembling molecules are mixed, the outcome is typically described in terms of co-assembly or self-sorting (Fig. 1). In this framework, self-sorting refers to the formation of separate supramolecular objects composed of a single molecular component, whereas co-assembly implies molecular-level mixing of both components within a shared supramolecular structure.^1–3^ While this binary distinction is widely used, it is incomplete. Self-sorting does not only occur between separate supramolecular objects, but can also arise within a co-assembled structure, leading to so-called “orthogonal” co-assembly, with spatial segregation of components across multiple length scales rather than complete macroscopic separation.

**Fig. 1:**
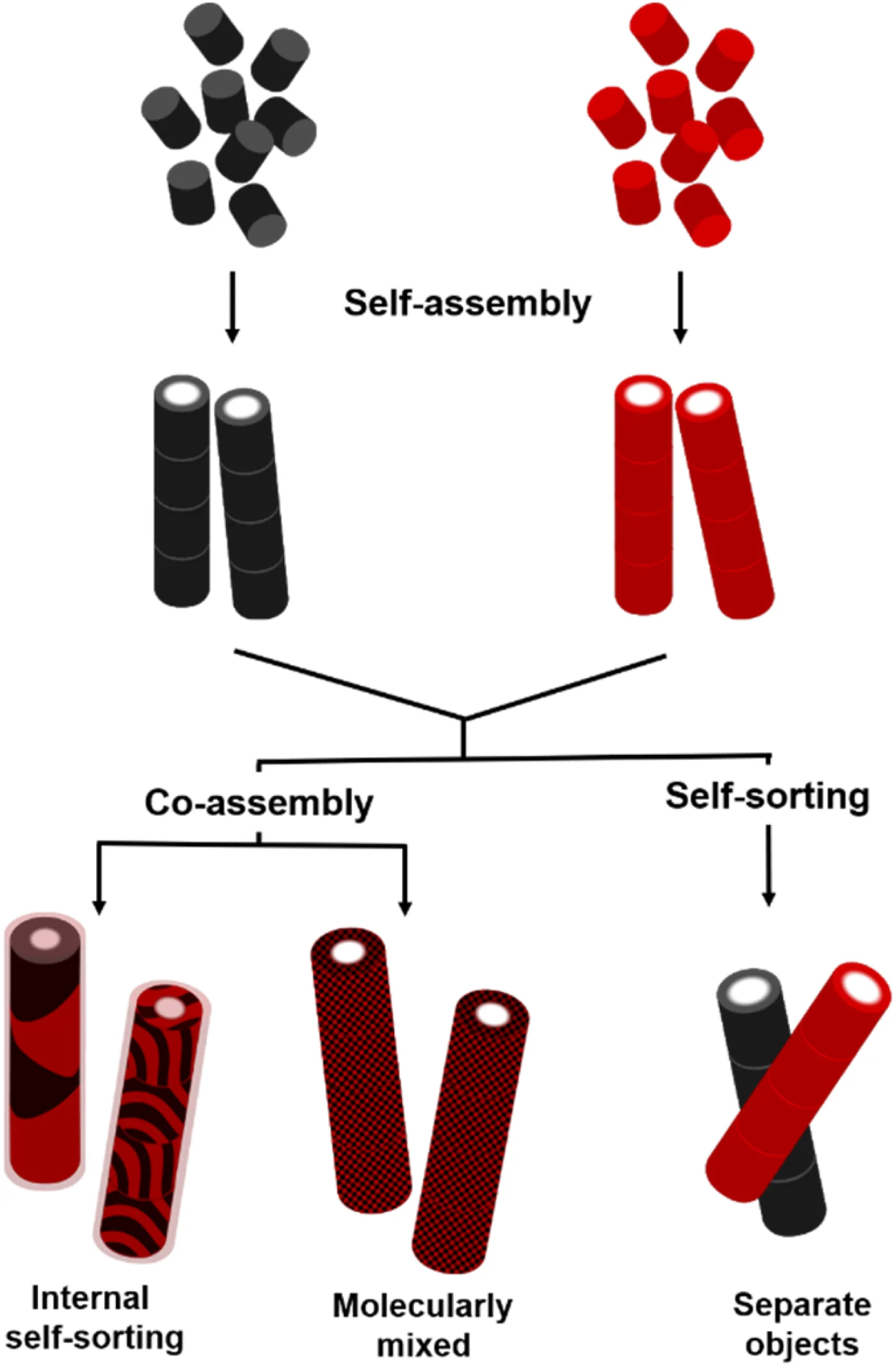
Schematic illustration of possible outcomes when two self-assembling molecules are mixed relevant to this work. Components may self-sort into separate supramolecular objects, co-assemble at the molecular level to form new structures or form single co-assembled structures in which the components remain spatially segregated across different length scales.

The outcome of multicomponent self-assembly therefore defines not only structure but also function. Different modes of organisation will lead to distinct intermolecular contacts and packing arrangements, which in turn determine material properties. For example, self-sorted fibre networks formed from two components have been proposed for optoelectronic applications, where the extent of contact between different species influences energy and electron transfer efficiency.^4,5^ Control over the mode of assembly is therefore essential yet remains difficult to achieve or predict.

Low molecular weight gelators (LMWGs) provide a useful platform to explore these questions. These small molecules self-assemble into one-dimensional structures such as fibres or nanotubes, which entangle to form networks that immobilise the solvent.^6–10^ Many multicomponent LMWG systems have been reported, with applications in cell culture, optoelectronics, and sensing.^5,11,12^ In most cases, however, the outcome is described only at the level of the primary structure, largely because resolving organisation across multiple length scales is experimentally challenging.

Furthermore, most studies focus on a single composition, even though multicomponent systems are not constrained to exhibit a single assembly mode across all ratios and concentrations. For example, we have previously shown that mixtures of diastereomeric dipeptides (l,l-2NapFF and l,d-2NapFF, Fig. 2) form two distinct nanotube populations across a range of compositions, with one population remaining invariant and the other varying systematically.^13^ In multicomponent systems, chirality is often invoked to rationalise whether enantiomeric or diastereomeric components co-assemble or self-sort.^14^ As a result, chirality is commonly framed as controlling a binary outcome between mixing and separation.^15–21^ For peptide systems, equimolar mixtures of enantiomers have been reported to often form co-assembled structures which exhibit a synergistic enhancement in material properties.^22–26^ Chirality has also been shown to strongly influence liquid–liquid phase separation in dipeptide coacervates.^27^ However, this perspective treats self-sorting primarily as a process occurring between different supramolecular objects. Much less attention has been paid to how chirality and composition together influence spatial organisation within a single co-assembled structure. Composition-dependent assembly has been recently reported for a pair of enantiomeric pentapeptides,^25^ where the enantiomers individually form twisted fibres and transition to micrometer-scale sheets at equimolar composition. At non-equimolar composition a mixture of fibres and sheets was instead demonstrated. It remains however unclear whether enantiomeric components that form structurally identical assemblies can generate a common supramolecular architecture and, particularly, whether the individual enantiomeric components molecularly co-assemble within the new structure or retain internal compositional segregation.

**Fig. 2:**
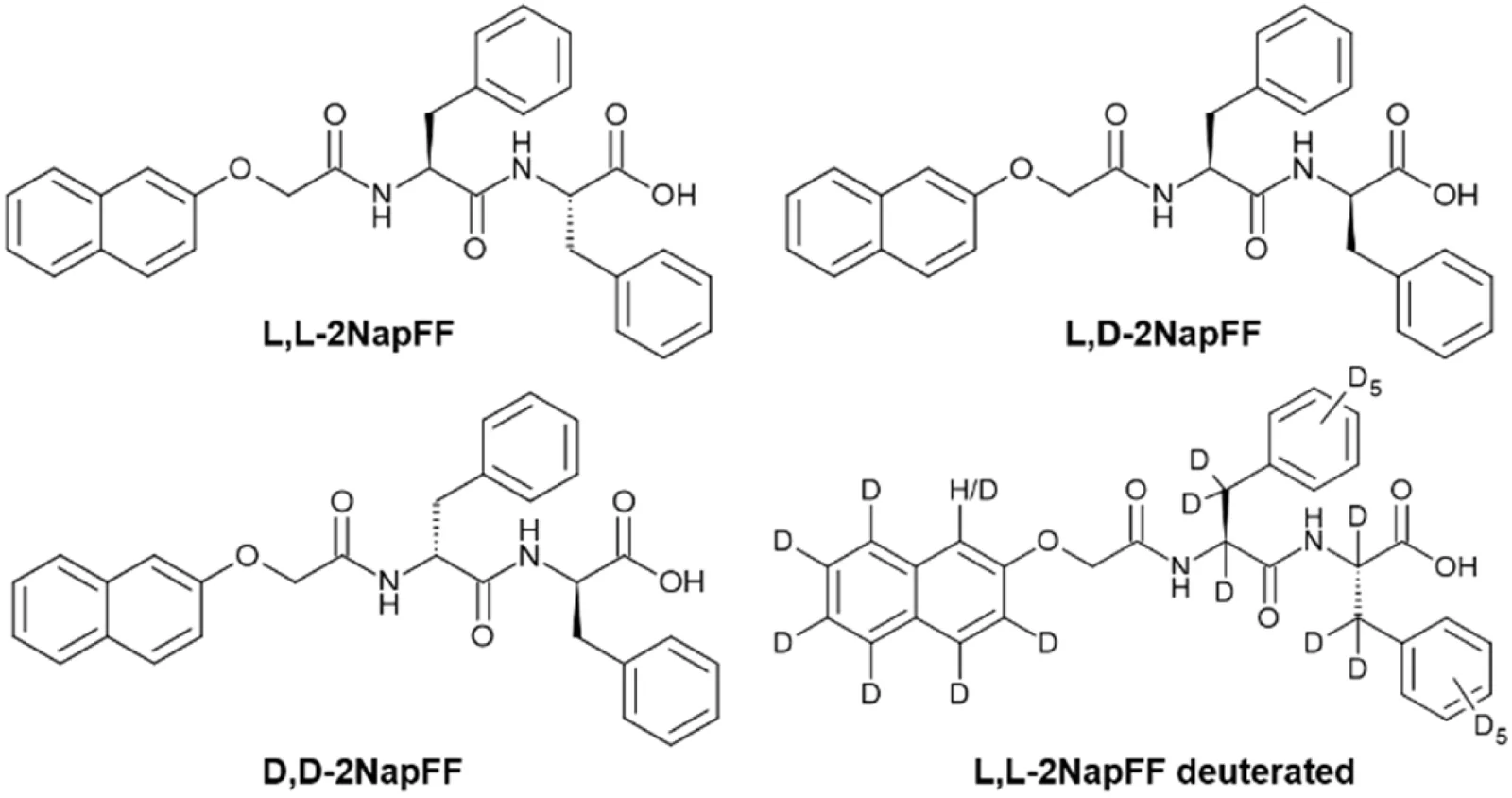
Chemical structures of the enantiomeric functionalised dipeptides l,l-2NapFF and d,d-2NapFF investigated in this work.

Here, we address this question using mixtures of enantiomeric dipeptides that independently form structurally identical nanotubes (l,l-2NapFF and d,d-2NapFF). By systematically varying composition, we show that these systems do not simply transition between co-assembly and self-sorting. Instead, we show that self-sorting can occur within a single supramolecular object while co-assembly defines the overall structure. This decouples molecular mixing from structural co-assembly and demonstrates that self-sorting can be an intrinsic feature of a co-assembled architecture operating across multiple length scales.

## Results and discussion

We have previously shown that l,l-2NapFF and d,d-2NapFF independently self-assemble at high pH (pH 10.5 at a concentration of 10 mg mL^−1^) to form nanotubes with an internal radius of 1.7 nm and a wall thickness of approximately 2 nm (Fig. 4g and Table S1).^15^ Cryo-EM analysis of l,l-2NapFF revealed the molecular packing at near-atomic resolution, where three distinct peptide conformations assemble into a protofilament, with eight protofilaments twisting to generate the hollow nanotube structure.^16^ These structural features are consistent with small-angle neutron scattering (SANS), where the fitted parameters closely match those derived from cryo-EM. The corresponding SANS data for d,d-2NapFF are effectively identical, confirming that both enantiomers form structurally equivalent nanotubes.^15^

We have also reported that an equimolar (50 : 50) mixture of l,l-2NapFF and d,d-2NapFF leads to the formation of a new structure that differs significantly from the self-sorted nanotubes observed for diastereomeric mixtures.^15^ This suggests that the enantiomers co-assemble under these conditions. However, the origin of this co-assembled structure and its dependence on composition remain unclear, particularly given that both components individually form identical assemblies.

To address this, we prepared mixtures of d,d-2NapFF and l,l-2NapFF at ratios of 75 : 25, 50 : 50, and 25 : 75 (expressed as d,d-2NapFF : l,l-2NapFF throughout). The resulting samples exhibit clear composition-dependent behaviour. Visually, the 50 : 50 mixture shows the highest turbidity (Fig. 3a), indicating the formation of structures distinct from those in the pure systems. This is supported by rheology, where all mixtures display higher viscosity than the individual components, consistent with the formation of more complex or interconnected structures (Fig. 3b).

**Fig. 3:**
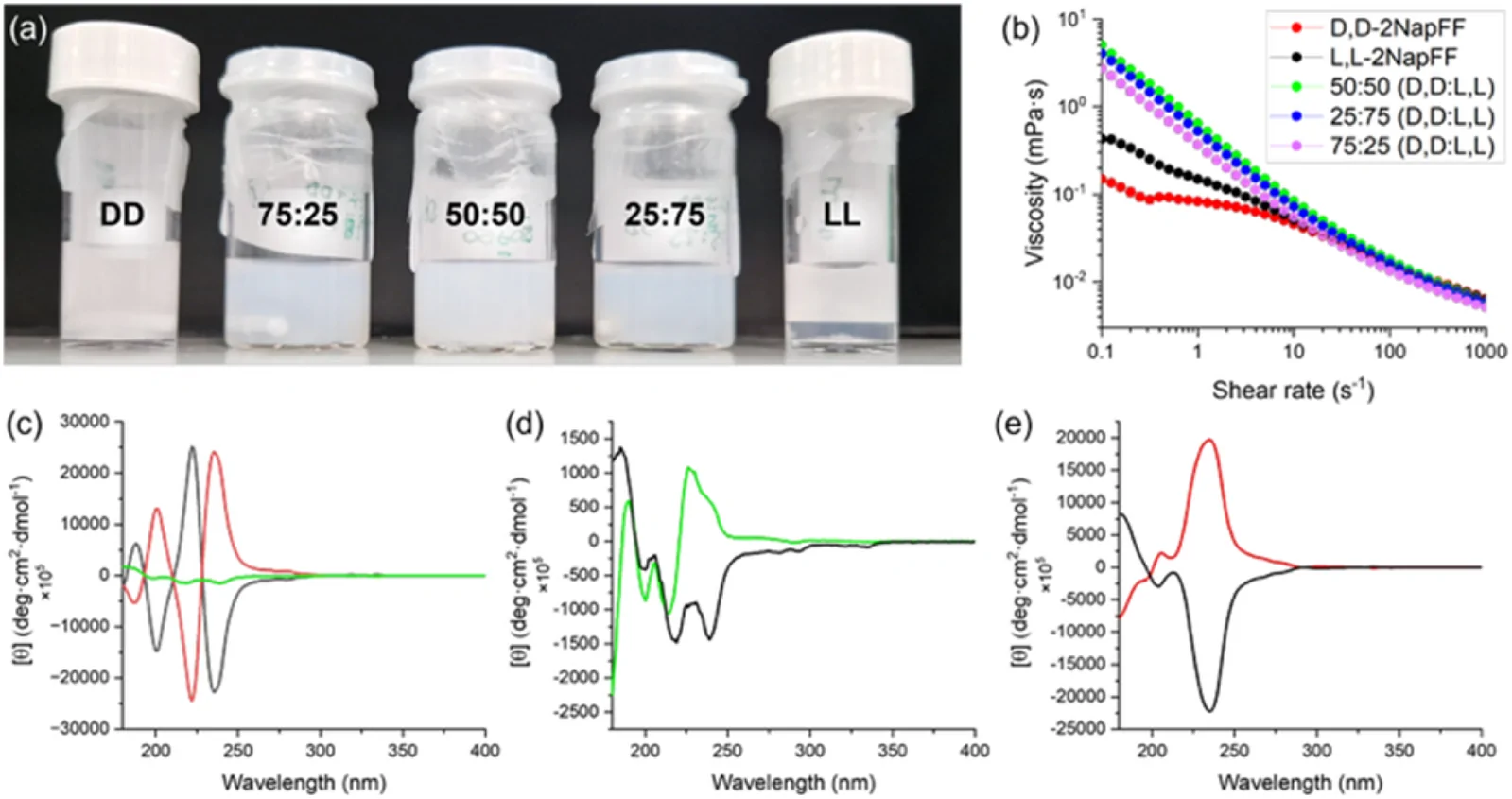
(a) Photographic images and (b) shear viscosity data of solutions of mixtures of d,d-2NapFF (red), l,l-2NapFF (black) and mixtures at ratios of 75 : 25 (purple), 50 : 50 (green) and 25 : 75 (blue). (c) CD data for solutions of d,d-2NapFF (red), l,l-2NapFF (black) and a 50 : 50 mixture (green) before inducing the self-assembly. (d) CD data of 50 : 50 mixture (black) compared to theoretical sum of the 1 : 1 spectrum of d,d-2NapFF and l,l-2NapFF (green) after self-assembly. (e) CD data for mixtures of d,d-2NapFF and l,l-2NapFF at ratios of 75 : 25 (red) and 25 : 75 (black) after self-assembly. All data were collected in D_2_O at pD 11.0 ± 0.1.

We note that the two enantiomers alone exhibit different turbidity and slightly different viscosity at low shear rates (Fig. 3a and b). As we have shown that both molecules form structurally equivalent nano-assemblies, we attribute these differences to higher-order aggregation rather than primary structure, leading to differences in light scattering and increased viscosity. However, the viscosities converge at higher shear rates, indicating that the equivalent primary structures undergo similar shear-thinning behaviour.

Circular dichroism (CD) spectroscopy provides further insight into the chiral organisation. The individual enantiomers exhibit mirror-image spectra (Fig. 3c), indicating the formation of structurally equivalent self-assemblies with opposite handedness. Upon assembly, the 50 : 50 mixture shows a markedly reduced CD signal (Fig. 3c), which would be expected if the contributions from each enantiomer cancel. However, the observed spectrum does not correspond to a simple linear combination of the two self-sorted systems (Fig. 3d), indicating that the structure formed is not a trivial superposition of independent assemblies. Instead, the data suggest the formation of a distinct chiral environment arising from the formation of a co-assembled structure incorporating both enantiomers. In this, we emphasise that the CD spectra are not just those of the molecule, but rather the assembled supramolecular structure and are therefore difficult to fully interpret. At off-stoichiometric compositions (75 : 25 and 25 : 75), the CD spectra more closely resemble those of the dominant enantiomer, with mirror-image behaviour depending on which component is in excess (Fig. 3e). This indicates that the overall chirality of the assembled structures is dictated by the major component, consistent with previous observations in related systems.^18^

Taken together, these data show that mixing the enantiomers leads to the formation of new assemblies whose structure and chiral properties depend strongly on composition. In particular, the 50 : 50 mixture appears to form a distinct co-assembled structure, while off-stoichiometric mixtures retain features of the dominant component, suggesting that both co-assembly and self-sorting processes are operating simultaneously and in a composition-dependent manner.

To directly investigate the nature of the co-assembled structure and probe how composition influences molecular packing and supramolecular organisation, we combined cryo-electron microscopy (cryo-EM) with small-angle neutron scattering (SANS). Cryo-EM provides real-space information on morphology and heterogeneity, while SANS defines the internal radial organisation of the assemblies. As expected, both l,l-2NapFF and d,d-2NapFF form thin nanotubes with indistinguishable morphology (Fig. 4a and b). Consistent with previous reports, near-atomic resolution analysis confirms that the molecular packing in d,d-2NapFF closely matches that of l,l-2NapFF (Fig. S5 and Table S8),^16^ reinforcing that the two enantiomers independently generate structurally equivalent assemblies with opposite handedness as indicated by the CD.

**Fig. 4:**
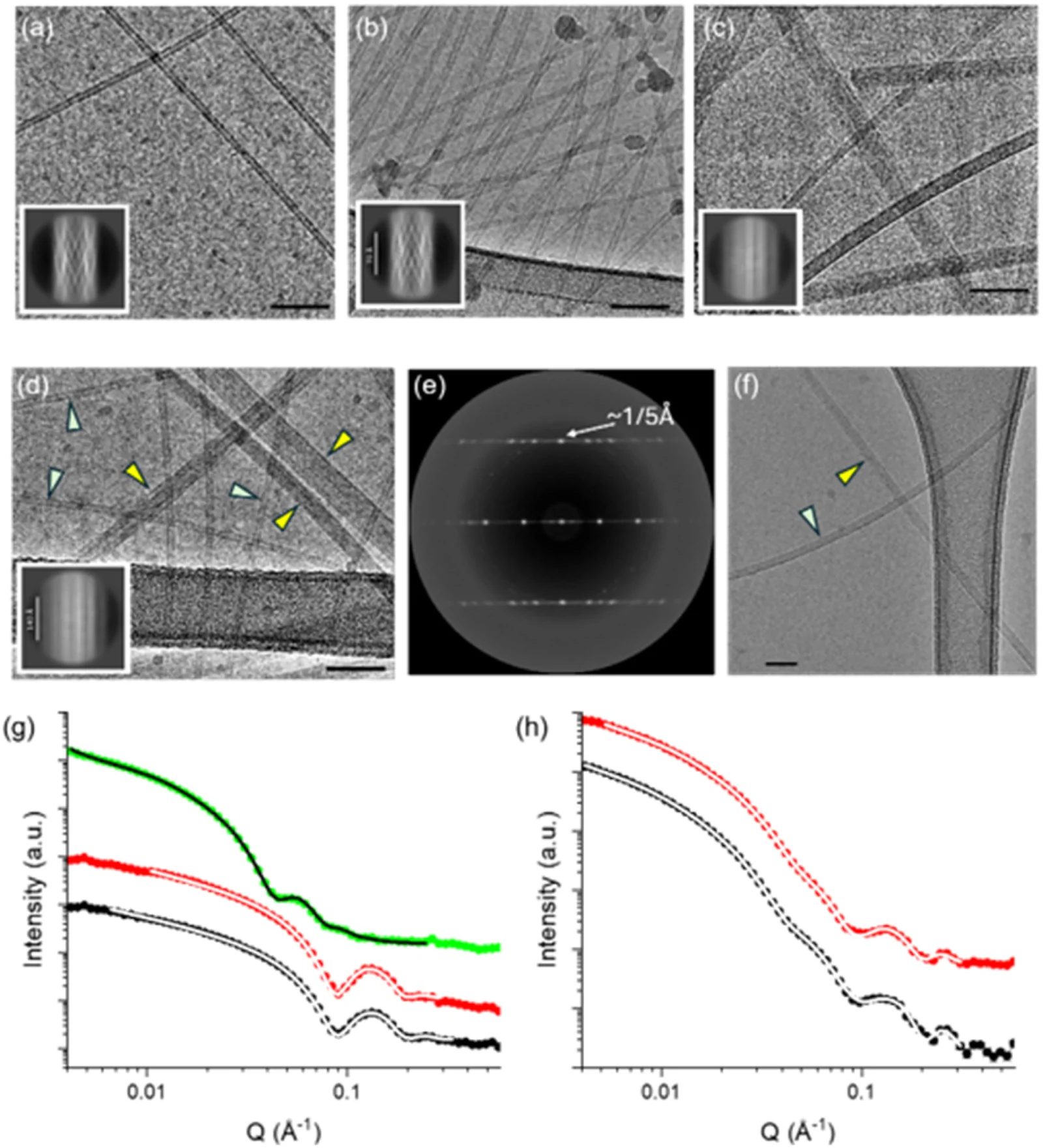
Representative cryo-EM micrograph of (a) l,l-2NapFF, (b) d,d-2NapFF, (c) 50 : 50 mixture (dd : ll), (d) 75 : 25 mixture (dd : ll) and (f) 25 : 75 mixture (dd : ll). 2D average of tube/bundle is shown as inset in the respective micrograph. Yellow arrowheads indicate structural populations of the 50 : 50 bundle with striations and green arrowheads indicate the individual self-sorted tubes. Scale bar in micrograph = ∼50 nm. (e) Averaged power spectrum generated from bundles shown in d. (g) SANS data for l,l-2NapFF (black), d,d-2NapFF (red) and 50 : 50 mixture (green). (h) SANS data for mixtures at a ratio of 25 : 75 (black) and 75 : 25 (red) (dd : ll). In all SANS data, solid lines represent model fits (Tables S1, S2 and S3 in SI).

In contrast, the 50 : 50 mixture forms a distinct population of heterogeneous structures with significantly larger diameters (Fig. 4c). These differ markedly from the individual nanotubes and are consistent with the formation of a bundled co-assembled structure. Striations are observed along the bundle axis, indicating lateral association of primary building blocks, and are reminiscent of nanowire-like features reported in related enantiomeric systems.^19^ At off-stoichiometric compositions (75 : 25 and 25 : 75), two distinct populations are observed: nanotubes matching the pure components and larger heterogeneous bundles analogous to those in the 50 : 50 sample (Fig. 4d and f). Due to the structural heterogeneity of the 50 : 50 bundle, broad structural populations of thinner and thicker bundles are present at these compositions. The cryo-EM micrographs are representative of areas where both distinct populations (bundles and nanotubes) are shown. This directly demonstrates that co-assembly only occurs at a specific composition, while excess material self-sorts to form nanotubes.

Although the structural heterogeneity and sample viscosity prevent full reconstruction of the bundled architecture, the averaged power spectrum reveals a clear layer line at ∼1/(5 Å), corresponding to an axial periodicity of approximately 5 Å (Fig. 4e). This spacing is consistent with peptide stacking along the longitudinal axis and indicates that the bundles retain a defined internal order despite their morphological variability. Together, the cryo-EM data show that composition controls not only whether co-assembly occurs, but notably results in the coexistence of co-assembled and self-sorted structures within the same system.

SANS measurements corroborate these observations and provide quantitative insight into the radial organisation of the enantiomers and the cross-sectional dimensions of the co-assembled structures. Both pure components are well described by hollow cylinder models with radii of ∼1.7 nm and wall thicknesses of 1.8–2.0 nm (Fig. 4g), in agreement with cryo-EM and previous reports.^15^ In contrast, the 50 : 50 mixture is best described by a larger flexible cylinder model with an effective radius of 8.9 nm combined with a power law, consistent with the presence of bundled structures. In agreement with the broad range of sizes shown in the cryo-EM, a polydispersity value of 0.1 was added to the radius to best capture the data, with the resulting effective radius being representative of the average cross-sections of all the bundle populations. Although this differs from earlier fits based on highly polydisperse hollow cylinders,^15^ both approaches converge on a substantially increased overall dimension, reflecting the same underlying structural transition.

Clearly, the bundled structures are intrinsically heterogeneous and evolve over time. SANS measurements on aged samples show a further increase in effective average radius of the bundled structures (Fig. S4 and Table S7), indicating continued structural reorganisation. This behaviour is consistent with a kinetically evolving assembly process rather than a single static structure. For off-stoichiometric mixtures, the scattering profiles are well described by a combination of hollow cylinder models (corresponding to self-sorted nanotubes) and the larger flexible cylinder model (corresponding to polydisperse co-assembled bundles) (Fig. 4h and Table S3). Together with the cryo-EM data, this confirms that the system resolves compositional imbalance by forming both co-assembled and self-sorted structures simultaneously.

Overall, these data establish that co-assembly in this system is stoichiometry-dependent and that the resulting structures are hierarchical, with co-assembled bundles coexisting with self-sorted nanotubes. The slight variations in fitted structural parameters likely reflect the complexity of the system and the presence of local ordering within the bundles, rather than fundamental changes to the primary nanotube structure.

To resolve the internal organisation of the co-assembled structure at a 50 : 50 composition, we employed contrast-matched SANS. Mixtures were prepared using d,d-2NapFF with a perdeuterated analogue of l,l-2NapFF (Fig. 1). In D_2_O, the scattering contrast of the deuterated component is significantly reduced relative to its hydrogenated analogue, such that the measured scattering arises predominantly from the d,d-2NapFF assemblies. This approach does not directly image compositional domains, but it enables selective interrogation of the spatial distribution of d,d-2NapFF within the co-assembled structure.

For both the 50 : 50 and 25 : 75 mixtures, the scattering profiles are highly similar (Fig. 5a and b), indicating that d,d-2NapFF adopts the same structural arrangement within both co-assemblies, while the excess l,l-2NapFF self-sorts into nanotubes, which do not scatter due to contrast-matching. This is consistent with the cryo-EM data, indicating the coexistence of an equimolar co-assembled bundle and self-sorted l,l-2NapFF nanotubes in the off-stoichiometric mixture.

**Fig. 5:**
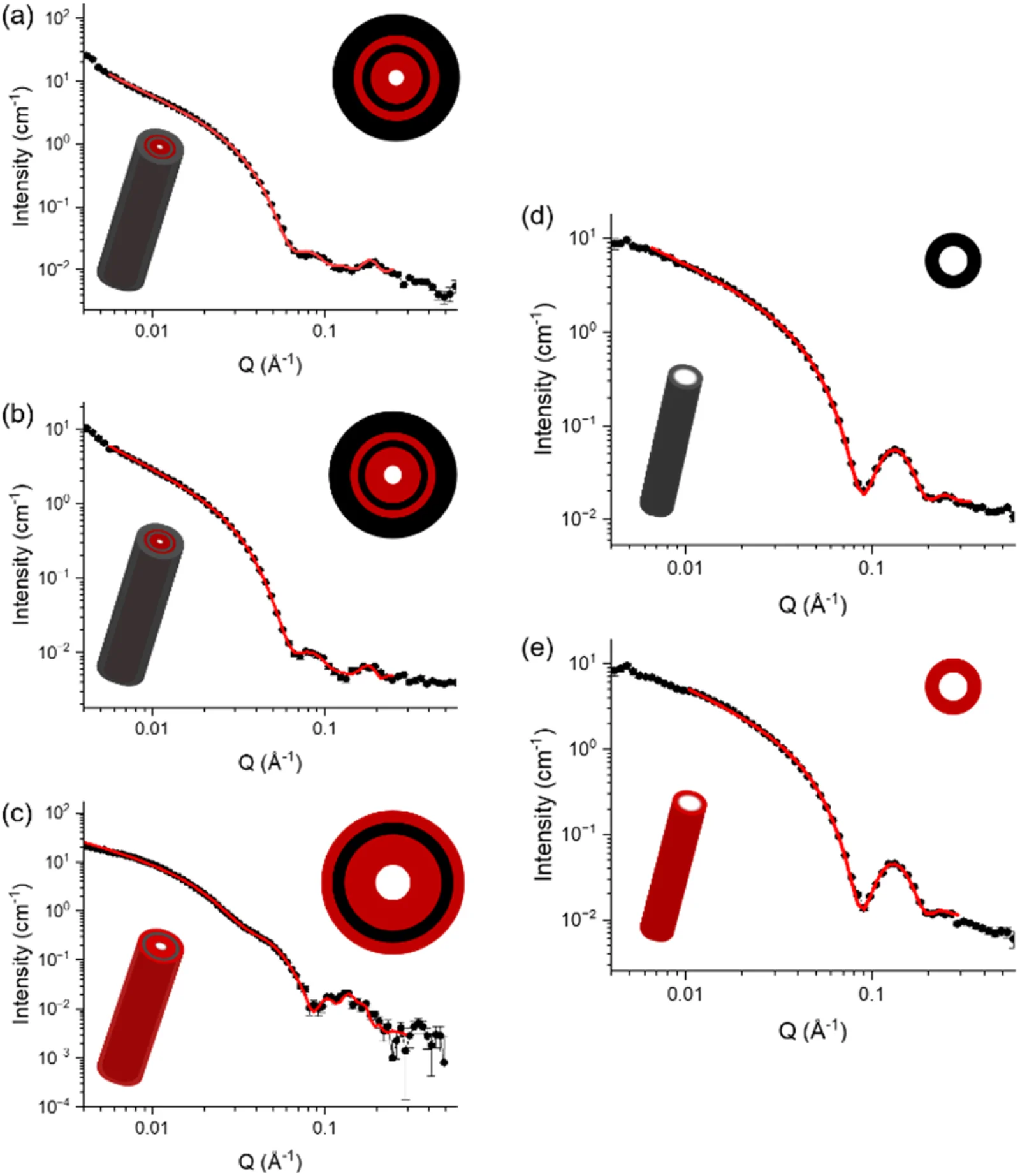
Contrast-matched SANS reveals internal self-sorting within the co-assembled structures. (a–c) Contrast-matched SANS data for mixtures of d,d-2NapFF and perdeuterated l,l-2NapFF at 50 : 50, 25 : 75, and 75 : 25 ratios. SANS data for individual solutions of (d) l,l-2NapFF and (e) d,d-2NapFF. Cartoon schematics of the fitted models are inset. Red regions indicate d,d-2NapFF and black regions indicate l,l-2NapFF. Model fits are shown as solid lines and detailed in the SI (Tables S1, S4 and S6, SI).

In contrast, the 75 : 25 mixture shows a distinct scattering profile (Fig. 5c), more closely resembling that obtained for the hydrogenated system. This indicates a change in internal organisation when d,d-2NapFF is present in excess, suggesting that composition influences not only the presence of co-assembly, but also the internal organisation of the resulting bundled structure.

All datasets exhibit a peak at ∼0.19 Å^−1^, showing that d,d-2NapFF adopts a repeated, locally ordered arrangement within the co-assembled structures. Importantly, this scattering is not consistent with simple molecular-level mixing of the two enantiomers. Instead, the data require spatially distinct regions containing d,d-2NapFF within the bundle. Several structural models were therefore considered to determine the simplest arrangements consistent with the contrast-matched scattering (Fig. S2, S3, Tables S5 and S6).

For the 50 : 50 and 25 : 75 compositions, the data are well described by a multi-walled cylindrical model, with similar fitted structural parameters for the two compositions. This model represents the scattering as arising from radially separated regions of d,d-2NapFF and is therefore consistent with internal self-sorting within the co-assembled bundle. A simpler flexible-cylinder model combined with a Lorentzian peak can also reproduce the principal features of these datasets (Fig. S2 and Table S5). Although this model does not uniquely define the real-space structure, it similarly requires a distinct radial region of d,d-2NapFF together with a periodic contribution. Thus, the detailed geometry of the domains cannot be uniquely determined from the SANS data, but both descriptions are consistent with spatial segregation of the enantiomers rather than homogeneous molecular-level mixing.

The 75 : 25 composition provides further evidence for this interpretation. Here, the scattering profile changes when d,d-2NapFF is present in excess, indicating a different internal organisation. A simpler model comprising flexible and hollow cylinders fails to reproduce the feature at ∼0.12 Å^−1^ (Fig. S3 and Table S6). This feature can instead be accounted for by contributions from the additional self-sorted d,d-2NapFF nanotubes observed by cryo-EM together with the repeated d,d-2NapFF regions within the co-assembled bundle. The contrast-matched SANS data therefore support a model in which d,d-2NapFF occupies compositionally distinct regions within the co-assembled structures, while excess peptide forms additional self-sorted nanotubes.

These results indicate that the identity of the excess component influences the radial arrangement within the co-assembled bundle, leading to compositional asymmetry across its cross-section. This behaviour is consistent with the CD data, where the dominant enantiomer dictates the overall chiral signature of the assembly.

We note that the use of a perdeuterated analogue may subtly influence assembly behaviour. While the key observation of internal segregation is robust, the precise assignment of layer identity should therefore be interpreted with caution. We note that we have previously shown that perdeuteration does not alter packing.^28^ Therefore, contrast-matched SANS provides evidence that the co-assembled structures are comprised by segregated sections of self-sorted enantiomer, with composition-dependent radial organisation.

Taken together, the combination of cryo-EM, SANS and contrast-matched SANS directly demonstrates the coexistence of self-sorted nanotubes and co-assembled bundles with internal self-sorting. Our data shows that enantiomers which self-assemble into structurally equivalent nanotubes undergo co-assembly upon mixing. The co-assembled structures are heterogeneous and larger in size, with a bundled morphology highlighted in the cryo-EM. Contrast-matched SANS reveals that the enantiomers within the single co-assembled bundle are internally organised into spatially segregated sections.

## Conclusions

In summary, we show that self-sorting can occur within a single supramolecular object while co-assembly defines the overall structure for a pair of enantiomeric functionalised dipeptides. Mixing the enantiomers leads to a form of co-assembly in which a single supramolecular structure is formed, yet the components remain spatially self-sorted within it. Further, co-assembly occurs in a stoichiometry-dependent manner, with co-assembling structures forming only at equimolar composition, while off-stoichiometric mixtures resolve compositional imbalance by forming the same co-assembled structure alongside self-sorted assemblies of the excess component. Contrast-matched neutron scattering demonstrates that this internal self-sorting gives rise to radially layered architectures rather than molecular-level mixing.

Although the energetic origins for the formation of the co-assembled bundle at equimolar composition are not yet identified, our work shows that co-assembly and self-sorting are not mutually exclusive but can coexist within a single supramolecular object across multiple length scales. We show that this behaviour is strongly dependent on molecular chirality and composition, with the diastereomeric pairs only undergoing self-sorting at a different composition.^13^ This study establishes a framework for understanding how composition affects multicomponent self-assembly and provides a route to exploit molecular chirality to influence hierarchical organisation for an enantiomeric peptide nanotube system. Stereochemistry has been recently shown to directly regulate higher-order assembly and LLPS in dipeptide systems, establishing principles for the design of synthetic protocells.^27^ We hypothesise that systems with closely related molecular packing preference may exhibit similar behaviour. Therefore, we believe that the results from this work will promote further investigations for the design of peptide systems with chirality-controlled self-assembly.

## Author contributions

Conceptualization: SB, DJA; synthesis of functionalised dipeptides: ASL; data curation: SB, RRS, JD; formal analysis: SB, RRS, LJM, DJA; funding acquisition: EHE, DJA; investigation: all; methodology: all; project administration: DJA; supervision: EHE, DJA; validation: LJM; visualization: SB, RRS; writing – original draft: SB, DJA; writing – review and editing: all.

## Conflicts of interest

There are no conflicts to declare.

## Supplementary Material

SC-017-D6SC04454A-s001

## Data Availability

The data supporting this article have been included as part of the supplementary information (SI). The authors have cited additional references within the SI.^29,30^ Supplementary information: CD and HT data; NMR spectra; SANS profiles with SasView fits and fitting parameters; cryo-EM reconstruction data. See DOI: https://doi.org/10.1039/d6sc04454a. The cryo-EM map of (D,D)-2NapFF is available at Electron Microscopy Data Bank (accession number: EMD-75070).

## References

[cit1] Buerkle L. E., Rowan S. J. (2012). Chem. Soc. Rev..

[cit2] Raeburn J., Adams D. J. (2015). Chem. Commun..

[cit3] Fleming S., Debnath S., Frederix P. W. J. M., Hunt N. T., Ulijn R. V. (2014). Biomacromolecules.

[cit4] Sugiyasu K., Kawano S.-i., Fujita N., Shinkai S. (2008). Chem. Mater..

[cit5] Cross E. R., Sproules S., Schweins R., Draper E. R., Adams D. J. (2018). J. Am. Chem. Soc..

[cit6] Chivers P. R. A., Smith D. K. (2019). Nat. Rev. Mater..

[cit7] Du X., Zhou J., Shi J., Xu B. (2015). Chem. Rev..

[cit8] Draper E. R., Adams D. J. (2017). Chem.

[cit9] Terech P., Weiss R. G. (1997). Chem. Rev..

[cit10] Weiss R. G. (2014). J. Am. Chem. Soc..

[cit11] Zhou M., Smith A. M., Das A. K., Hodson N. W., Collins R. F., Ulijn R. V., Gough J. E. (2009). Biomaterials.

[cit12] Makam P., Gazit E. (2018). Chem. Soc. Rev..

[cit13] Guan Q., McAulay K., Xu T., Rogers S. E., Edwards-Gayle C., Schweins R., Cui H., Seddon A. M., Adams D. J. (2023). Biomacromolecules.

[cit14] Basak S., Singh I., Ferranco A., Syed J., Kraatz H.-B. (2017). Angew. Chem., Int. Ed..

[cit15] McAulay K., Dietrich B., Su H., Scott M. T., Rogers S., Al-Hilaly Y. K., Cui H., Serpell L. C., Seddon A. M., Draper E. R., Adams D. J. (2019). Chem. Sci..

[cit16] Sonani R. R., Bianco S., Dietrich B., Doutch J., Draper E. R., Adams D. J., Egelman E. H. (2024). Cell Rep. Phys. Sci..

[cit17] Swanekamp R. J., DiMaio J. T. M., Bowerman C. J., Nilsson B. L. (2012). J. Am. Chem. Soc..

[cit18] Zhu X., Li Y., Duan P., Liu M. (2010). Chem.–Eur. J..

[cit19] Guo Z., Song Y., Wang Y., Tan T., Ji Y., Zhang G., Hu J., Zhang Y. (2021). Front. Mol. Biosci..

[cit20] Urban J. M., Ho J., Piester G., Fu R., Nilsson B. L. (2019). Molecules.

[cit21] Koga T., Matsuoka M., Higashi N. (2005). J. Am. Chem. Soc..

[cit22] Nagy-Smith K., Beltramo P. J., Moore E., Tycko R., Furst E. M., Schneider J. P. (2017). ACS Cent. Sci..

[cit23] Nagy K. J., Giano M. C., Jin A., Pochan D. J., Schneider J. P. (2011). J. Am. Chem. Soc..

[cit24] Wadai H., Yamaguchi K.-i., Takahashi S., Kanno T., Kawai T., Naiki H., Goto Y. (2004). Biochemistry.

[cit25] Duti I. J., Florian J. R., Kittel A. R., Amelung C. D., Gray V. P., Lampe K. J., Letteri R. A. (2023). J. Am. Chem. Soc..

[cit26] Swanekamp R. J., Welch J. J., Nilsson B. L. (2014). Chem. Commun..

[cit27] Peng S., Zhang X., Shi X.-L., Yu M., Cao S., Gao N. (2026). Angew. Chem., Int. Ed..

[cit28] Draper E. R., Dietrich B., McAulay K., Brasnett C., Abdizadeh H., Patmanidis I., Marrink S. J., Su H., Cui H., Schweins R., Seddon A., Adams D. J. (2020). Matter.

[cit29] Liu R., Zhang R., Li L., Kochovski Z., Yao L., Nieh M.-P., Lu Y., Shi T., Chen G. (2021). J. Am. Chem. Soc..

[cit30] Mollica A., Pinnen F., Stefanucci A., Mannina L., Sobolev A. P., Lucente G., Davis P., Lai J., Ma S.-W., Porreca F., Hruby V. J. (2012). J. Med. Chem..

