# Supplementary material for: Co-assembly and self-sorting are not mutually exclusive in enantiomeric peptide systems
Source: Chem Sci. 2026 Aug 27;17(41):19860–5. doi: 10.1039/d6sc04454a (PMC13541071; doi:10.1039/d6sc04454a)
Supplement: Supplementary file 1 [file SC-017-D6SC04454A-s001.pdf]

This journal is © The Royal Society of Chemistry 2026

# Supporting Information

## 1. Materials and methods

### 1.1 Materials

**L,L-2NapFF** and **D,D-2NapFF** were synthesized as previously reported; analytical data for the exact batches used here are below in Section 3.[19] The deuterated analogue of **L,L-2NapFF** was synthesized as previously reported; the same batch was used here as in the original report.[19] All other reagents and solvents were purchased from Sigma-Aldrich and Alfa Aesar and used as received without further purification. $D_2O$ (Sigma-Aldrich, 99.9 atom % D) was used throughout.

### 1.2 Solution preparation

To minimize any preparation artefacts, solutions were prepared in $D_2O$ throughout this work. To prepare solutions of **L,L-2NapFF** and **D,D-2NapFF**, the functionalized dipeptides were suspended in $D_2O$ and 1 molar equivalent of 0.1 M NaOD (diluted in $D_2O$ using the commercially available 40 wt % solution, Sigma-Aldrich) to achieve a final concentration of 10 mg/mL. The solutions were stirred overnight at 1000 rpm or until dissolution using a magnetic stirrer. The pD of the solution was adjusted up to 11.0 ± 0.1 using 1 M NaOD (diluted in $D_2O$ using the commercially available 40 wt % solution, Sigma-Aldrich) or 2 M DCl (diluted in $D_2O$ using the commercially available 35 wt % solution, Sigma-Aldrich) as necessary. During pD adjustment, the solutions were stirred at 1000 rpm to ensure homogeneous mixing of the acid or base and minimising stirring effects at different speeds.

To prepare the multicomponent solutions, pD adjusted solutions of each dipeptide were first prepared according to the protocol shown above at concentrations of 10 mg/mL. After pD adjustment, the solutions were mixed at volume ratios of 75:25, 50:50 and 25:75 using a pipette to achieve a total final concentration of 10 mg/mL. The solutions were allowed to stir at 1000 rpm overnight to ensure homogeneous mixing and avoid any reproducibility issues for the formation of multicomponent structures. If necessary, the pD of the solution was re-adjusted to 11.0 ± 0.1 using 1 M NaOD.

### 1.3 Rheological measurements

Viscosity measurements were performed on an Anton Paar Physica MCR101 rheometer using a cone and plate system (CP50, cone angle 1°) at a measuring gap of 0.101 mm. All samples were carefully poured on the bottom plate to minimize shear-induced effects. The data were collected at shear rates from 0.1 $s^{-1}$ to 1000 $s^{-1}$.

### 1.4 Small Angle Neutron Scattering (SANS)

SANS experiments were performed using the ZOOM and SANS2d instruments at the ISIS Neutron and Muon source of the STFC Rutherford Appleton Laboratory (Didcot, UK), experiment numbers RB2420098 and RB2420026. The samples were measured using a temperature-controlled sample changer and a water bath at 25°C. For both instruments, a

sample-to-detector distance of 4 m was employed. Samples were measured using neutrons of wavelengths spanning 1.75 – 16.5 Å to access a q range of 0.0042 – 0.77 $Å^{-1}$ at ZOOM and a q range of 0.0042 – 0.97 $Å^{-1}$ at SANS2D, where $q = 4\pi\sin(\theta/2)/\lambda$ and $\theta$ is the scattering angle. All solutions were measured in UV-spectrophotometer-grade quartz cuvettes (Hellma) with 2 mm path length. Scattering data was collected for 30 minutes for most solutions, while solutions containing the deuterated analogue were measured for 60 minutes to improve statistics. The 2D scattering patterns were azimuthally integrated to obtain 1D plots of intensity vs. Q using the facility-provided software. The scattering from the $D_2O$ background and the empty cell were subtracted from the gelator solutions using MantidPlot, and SANS curves were fitted using SasView (v.6.1.0), http://www.sasview.org/).

### 1.5 Circular Dichroism

Circular dichroism data were acquired on a Chirascan VX spectrometer (Applied Photophysics) using a quartz cuvette with a 0.01 mm path length. The spectra were collected in the range 180–400 nm with a scanning step size of 1.0 nm and scanning rate of 0.25 s at room temperature. The samples were prepared in Sterilin vials as described, keeping the same volumes of the components. Small amounts of the solutions were then transferred to the cuvette before measurement.

### 1.6 Cryo-EM

~3 µL of sample was applied to glow-discharged lacey carbon grids (Ted Pella Inc.). Excess sample was blotted using Whatman filter paper, and grids having a thin film of sample were plunge-frozen in liquid ethane using a Vitrobot (FEI). Frozen grids were subsequently imaged on a Glacios transmission electron microscope (Thermo Fisher Scientific) operated at 200 keV.

For **D,D-2NapFF**, ~700 dose-fractionated movies were collected, with a total electron dose of ~50 $e^{-}$ $Å^{-2}$ per movie, at a calibrated pixel size of 0.662 Å using a 300 keV Titan Krios transmission electron microscope (Thermo Fisher Scientific). Data processing was performed in cryoSPARC. Movie frames were motion-corrected using the Patch Motion Correction job, and contrast transfer function (CTF) parameters were estimated using Patch CTF Estimation. **D,D-2NapFF** tubular filaments were initially manually boxed to generate templates, which were subsequently used for automated particle picking with the Filament Tracer. False or poor-quality picks were removed through iterative 2D classification. A total of ~287,207 filament segments were selected for helical reconstruction using the Helix Refine job. Local CTF refinement was performed iteratively in conjunction with helical refinement to improve per-segment CTF parameters, resulting in the final reconstructed map.

## 2. Results

### 2.1 CD

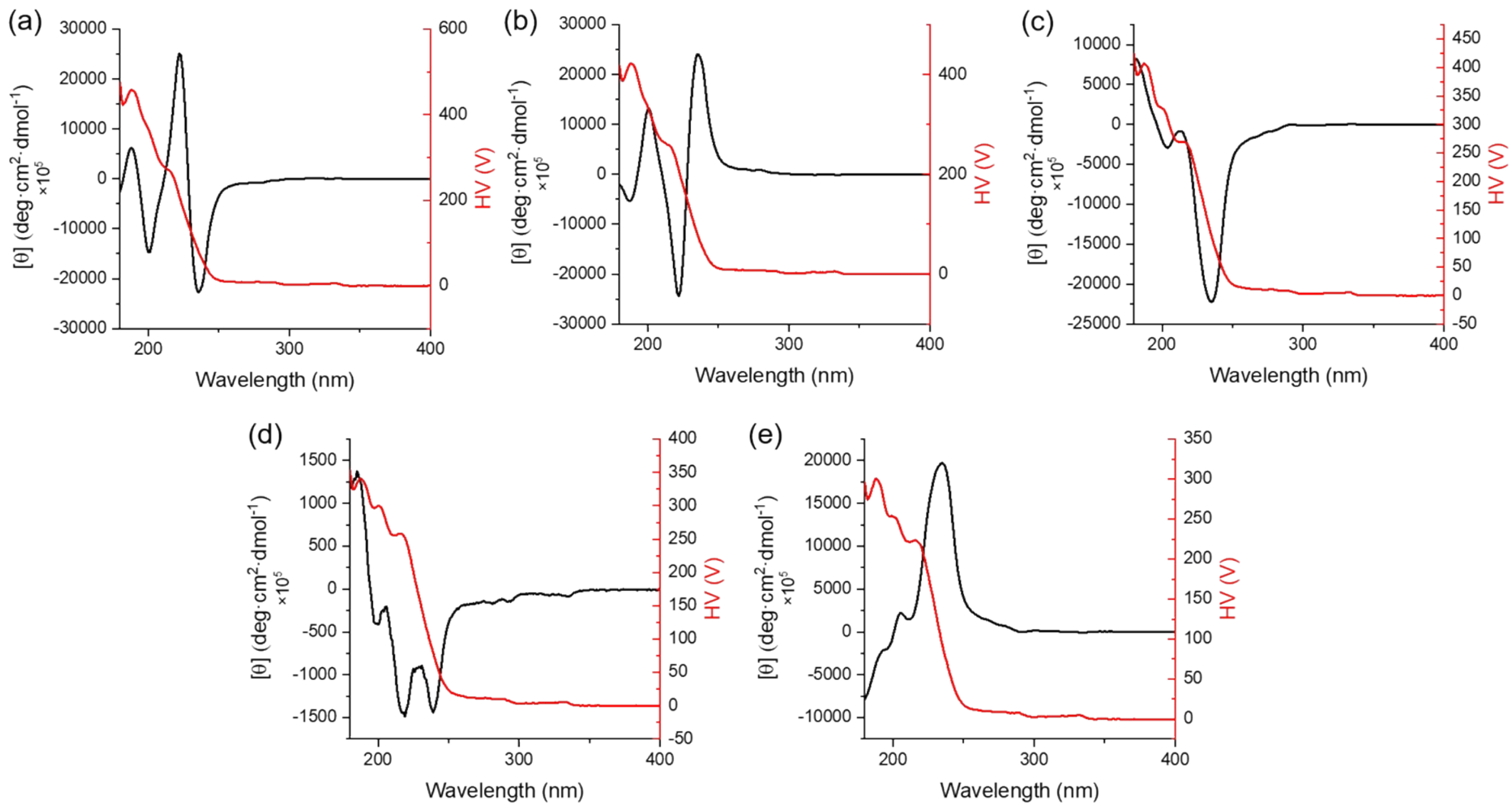

**Figure S1.** CD (black) and HT (red) data for solutions of (a) **L,L-2NapFF**, (b) **D,D-2NapFF**, (c) 25:75 (DD:LL) mixture, (d) 50:50 (DD:LL) mixture and (e) 75:25 (DD:LL) mixture.

## 2.2 SANS

**Table S1.** Fitting parameters for SANS data in Figure 4g (black and red). The data was fit to a hollow cylinder model. Values that were manually added and fixed have been labelled with *.

| Hollow Cylinder | L,L-2NapFF | | D,D-2NapFF | |
|---|---|---|---|---|
| | Value | Error | Value | Error |
| Scale | 0.005 | 5.12 x $10^{-5}$ | 0.004 | 4.19 x $10^{-5}$ |
| Background | 0.016 | 0.0002 | 0.012 | 0.0002 |
| Radius (Å) | 17.71 | 0.12 | 16.75 | 0.14 |
| Thickness (Å) | 17.88 | 0.22 | 19.88 | 0.233 |
| Length (Å) | 947.42 | 69.093 | 7068 | 1647.43 |
| $\chi^2$ | 4.07 | / | 8.17 | / |

**Table S2.** Fitting parameters for SANS data in Figure 4g (green). The data was fit to a flexible cylinder model combined with a power law. Values that were manually added and fixed have been labelled with *.

| **Flexible cylinder (A) + power law (B)** | **50:50 (DD:LL)** | |
|---|---|---|
| | **Value** | **Error** |
| **Background** | 0.015* | / |
| **A_scale** | 0.004 | $2.45 \times 10^{-5}$ |
| **A_radius (Å)** | 89.85 | 0.12 |
| **Radius polydispersity** | 0.1* | / |
| **A_kuhn_length (Å)** | 246.33 | 4.61 |
| **A_length (Å)** | 765.13 | 10.35 |
| **B_scale** | $3.27 \times 10^{-6}$ | $2.16 \times 10^{-7}$ |
| **B_power** | 3.07 | 0.015 |
| $\chi^2$ | 7.65 | |

**Table S3.** Fitting parameters for SANS data in Figure 4h. The data was fit to a flexible cylinder model combined with a power law and a hollow cylinder model. Values that were manually added and fixed have been labelled with *.

| Flexible cylinder (A) + power law (B) + Hollow cylinder (C) | 75:25 (DD:LL) | | 25:75 (DD:LL) | |
|---|---|---|---|---|
| | Value | Error | Value | Error |
| Background | 0.006* | / | 0.002* | |
| A_scale | 0.002 | $3.60 \times 10^{-5}$ | 0.002 | $3.80 \times 10^{-5}$ |
| A_radius (Å) | 76.53 | 0.37 | 86.53 | 0.26 |
| Radius polydispersity | 0.2* | / | 0.2* | / |
| A_kuhn_length (Å) | 840.12 | 10.62 | 175.37 | 6.43 |
| A_length (Å) | 209.96 | 23.68 | 1400 | 45.8 |
| B_scale | $2.08 \times 10^{-6}$ | $3.82 \times 10^{-7}$ | $6.15 \times 10^{-6}$ | $6.34 \times 10^{-7}$ |
| B_power | 3.02 | 0.037 | 2.84 | 0.03 |
| C_scale | 0.0017 | $5.25 \times 10^{-5}$ | 0.0014 | $8.79 \times 10^{-5}$ |
| C_radius (Å) | 20.46 | 0.26 | 21.57 | 0.40 |
| C_thickness (Å) | 12.86 | 0.48 | 10.1 | 0.74 |
| C_length (Å) | 8951.8 | 324.3 | 9149.3 | 628.12 |
| $\chi^2$ | 3.97 | | 2.83 | |

**Table S4.** Fitting parameters for SANS data in Figures 5a and 5b. The data was fit to a multi walled cylinder combined with a power law. Values that were manually added and fixed have been labelled with *.

| **Multi-walled cylinder (A) + power law (B)** | **50:50 (DD:dLL)** | | **25:75 (DD:dLL)** | |
|---|---|---|---|---|
| | **Value** | **Error** | **Value** | **Error** |
| **Background** | 0.007 | $2.95 \times 10^{-4}$ | 0.004 | $2.32 \times 10^{-4}$ |
| **A_scale** | 0.002 | $5.69 \times 10^{-5}$ | 0.001 | $1.05 \times 10^{-4}$ |
| **A_radius_core (Å)** | 9.447 | 0.51 | 11.43 | 1.41 |
| **A_length (Å)** | 4714.6 | 579.7 | 4832.1 | 585.3 |
| **A_thickness_shell_1 (Å)** | 26* | / | 27.02 | 1.74 |
| **A_thickness_shell_2 (Å)** | 11.33 | 0.48 | 10.26 | 0.81 |
| **A_thickness_shell_3 (Å)** | 12.61 | 0.38 | 10.82 | 0.80 |
| **B_scale** | $2.28 \times 10^{-5}$ | $3.72 \times 10^{-6}$ | $1.66 \times 10^{-5}$ | $3.89 \times 10^{-6}$ |
| **B_power** | 2.30 | 0.036 | 2.14 | 0.06 |
| **$\chi^2$** | 4.85 | | 1.66 | |

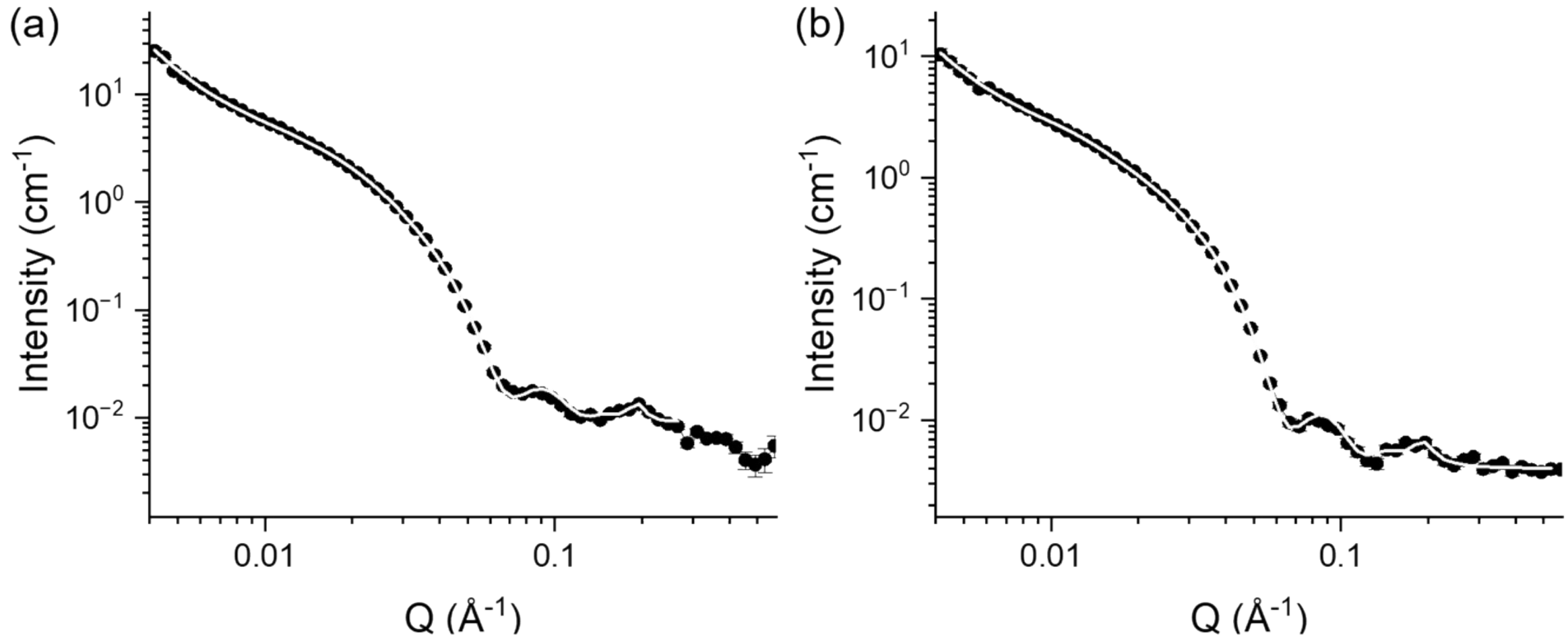


**Figure S2.** Contrast matched SANS data obtained for a mixture of **D,D-2NapFF** and deuterated **L,L-2NapFF** at a ratio of 50:50 and 25:75 fitted to a flexible cylinder combined with a power law and a Lorentzian peak. The model fits the data well, suggesting that **D,D-2NapFF** assembles in the co-assembled structure to form cylinders with smaller radii in a repeated arrangement.

**Table S5.** Fitting parameters for SANS data in Figure S2. The data was fit to a flexible cylinder model combined with a power law and a Lorentzian peak. Values that were manually added and fixed have been labelled with *.

| Flexible cylinder (A) + power law (B) + Lorentzian peak (C) | 50:50 (DD:dLL) | | 25:75 (DD:dLL) | |
|---|---|---|---|---|
| | **Value** | **Error** | **Value** | **Error** |
| **Background** | 0.009* | / | 0.004* | / |
| **A_scale** | 0.0008 | $2.22 \times 10^{-5}$ | 0.0006 | $4.62 \times 10^{-6}$ |
| **A_radius (Å)** | 55.23 | 0.32 | 58.21 | 0.20 |
| **A_kuhn_length (Å)** | 131.65 | 9.3 | 278.33 | 12.39 |
| **A_length (Å)** | 471.41 | 16.59 | 361.94 | 10.99 |
| **B_scale** | $3.35 \times 10^{-6}$ | $5.41 \times 10^{-7}$ | $2.91 \times 10^{-6}$ | $4.90 \times 10^{-7}$ |
| **B_power** | 2.81 | 0.03 | 2.67 | 0.03 |
| **C_scale** | 0.006 | 0.002 | 0.002 | 0.0004 |
| **C_peak_pos** | 0.19* | / | 0.19* | / |
| **C_peak_hwhm** | 0.008 | 0.003 | 0.02 | 0.005 |
| **$\chi^2$** | 1.85 | | 1.37 | |

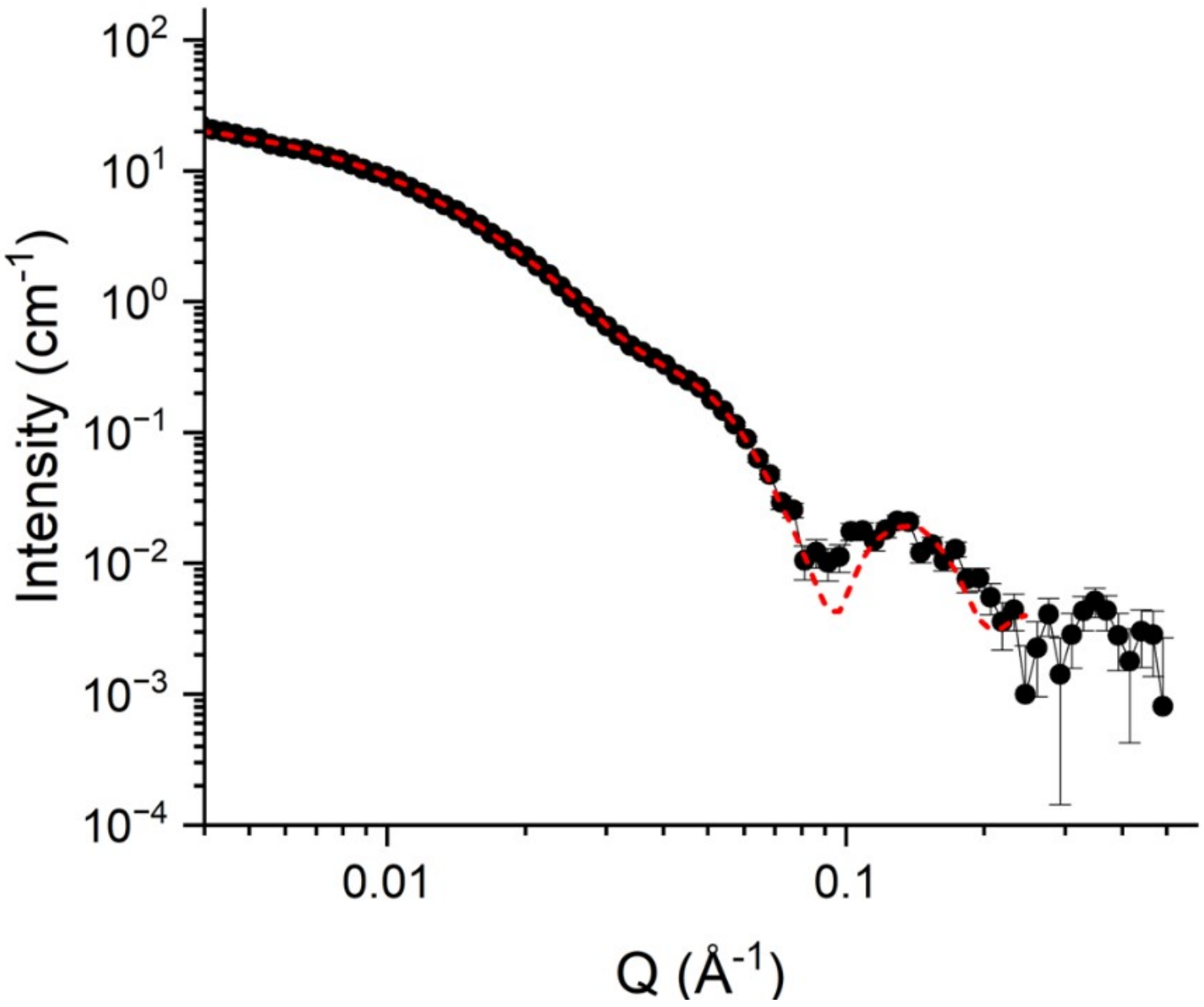


**Figure S3.** Contrast matched SANS data obtained for a mixture of **D,D-2NapFF** and deuterated **L,L-2NapFF** at a ratio of 75:25 fitted to a flexible cylinder combined with a hollow cylinder model. The fitted model is in red to highlight how the fit does not fully capture the behaviour around 0.12 Å$^{-1}$.

**Table S6.** Fitting parameters for SANS data in Figures 5c and Figure S3. The data was fit to either a multi walled cylinder combined with a hollow cylinder or to a flexible cylinder combined with a hollow cylinder. Values that were manually added and fixed have been labelled with *.

| Multi-walled cylinder (A) + hollow cylinder (B) | 75:25 (DD:dLL) | | Flexible cylinder (A) + hollow cylinder (B) | 75:25 (DD:dLL) | |
|---|---|---|---|---|---|
| | Value | Error | | Value | Error |
| Background | 0.003* | / | Background | 0.003* | / |
| A_scale | 0.0012 | $9.62 \times 10^{-5}$ | A_scale | 0.0004 | $5.06 \times 10^{-6}$ |
| A_radius_core (Å) | 23.95 | 2.14 | A_radius (Å) | 102.07 | 0.89 |
| A_length (Å) | 5000* | / | A_kuhn_length (Å) | 210.19 | 0.006 |
| A_thickness_shell_1 (Å) | 45.29 | 2.4 | A_length (Å) | 840.81 | 23.93 |
| A_thickness_shell_2 (Å) | 17.48 | 1.24 | | | |
| A_thickness_shell_3 (Å) | 17.81 | 1.29 | | | |
| B_scale | 0.0018 | $8.14 \times 10^{-5}$ | B_scale | 0.002 | $8.84 \times 10^{-5}$ |
| B_radius (Å) | 15.24 | 0.62 | B_radius (Å) | 16.82 | 0.53 |
| B_thickness (Å) | 19.34 | 0.97 | B_thickness (Å) | 17.71 | 0.88 |
| B_length (Å) | 4000* | / | B_length (Å) | 4000* | / |
| $\chi^2$ | 5.68 | | $\chi^2$ | 2.50 | |

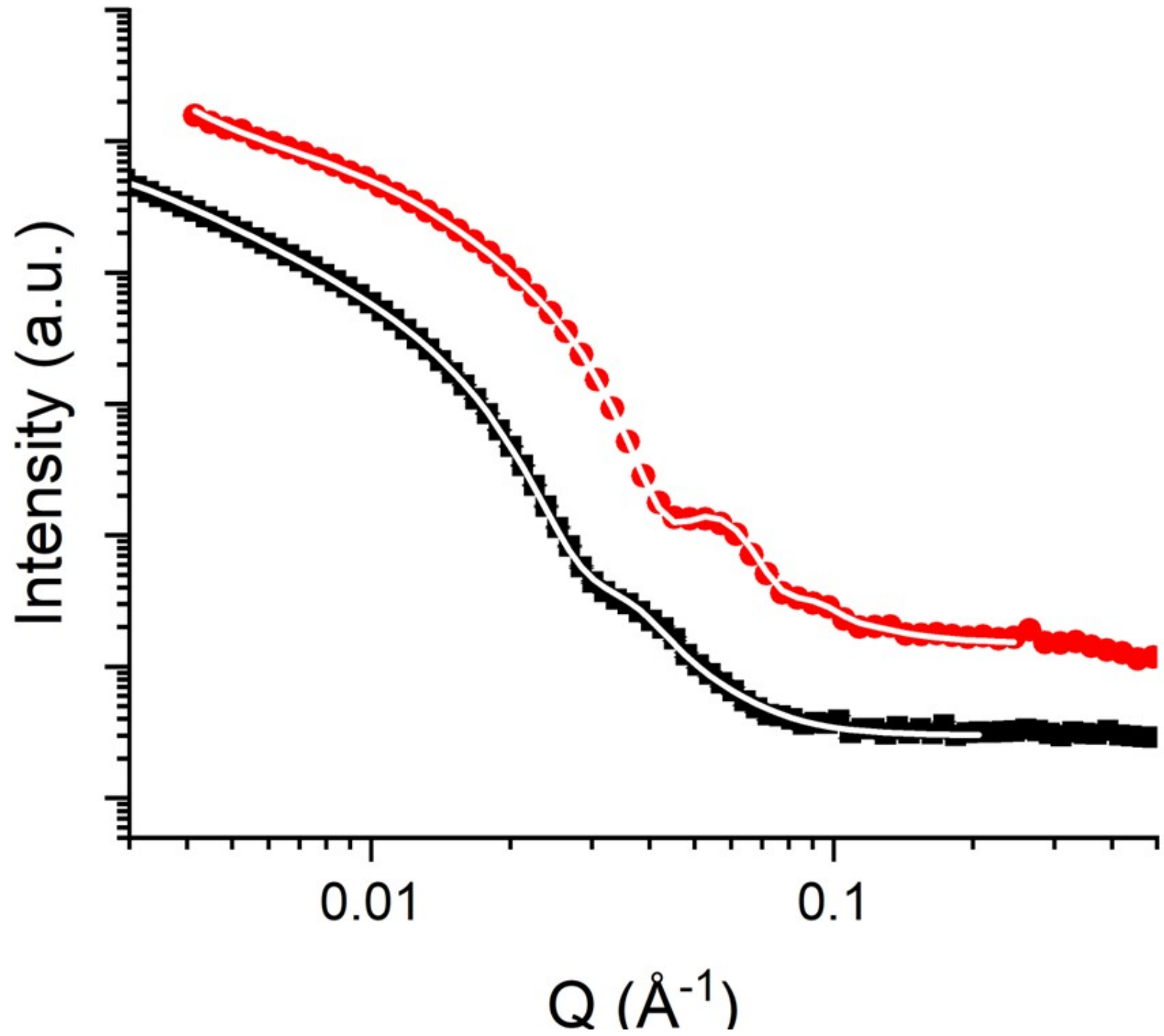


**Figure S4.** SANS data obtained for a mixture of **D,D-2NapFF** and **L,L-2NapFF** at a ratio of 50:50 after 1 day (red, same data as Figure 4g, green) and after 1 year (black). The same sample was used for both measurements. The aged data was fit to a flexible cylinder model.

**Table S7.** Fitting parameters for SANS data in Figure S4. The data was fit to a flexible cylinder model. Values that were manually added and fixed have been labelled with *.

| Flexible cylinder | 50:50 (DD:LL) aged (same sample) | |
|---|---|---|
| | Value | Error |
| Background | 0.037 | 1.26 x $10^{-5}$ |
| Scale | 0.03* | / |
| Radius (Å) | 133.56 | 0.23 |
| Radius polydispersity | 0.2* | / |
| Kuhn Length (Å) | 490.53 | 7.58 |
| Length (Å) | 3493.1 | 54.76 |
| $\chi^2$ | 7.26 | |

## 2.3 Cryo-EM reconstruction of tube formed by D,D-2NapFF

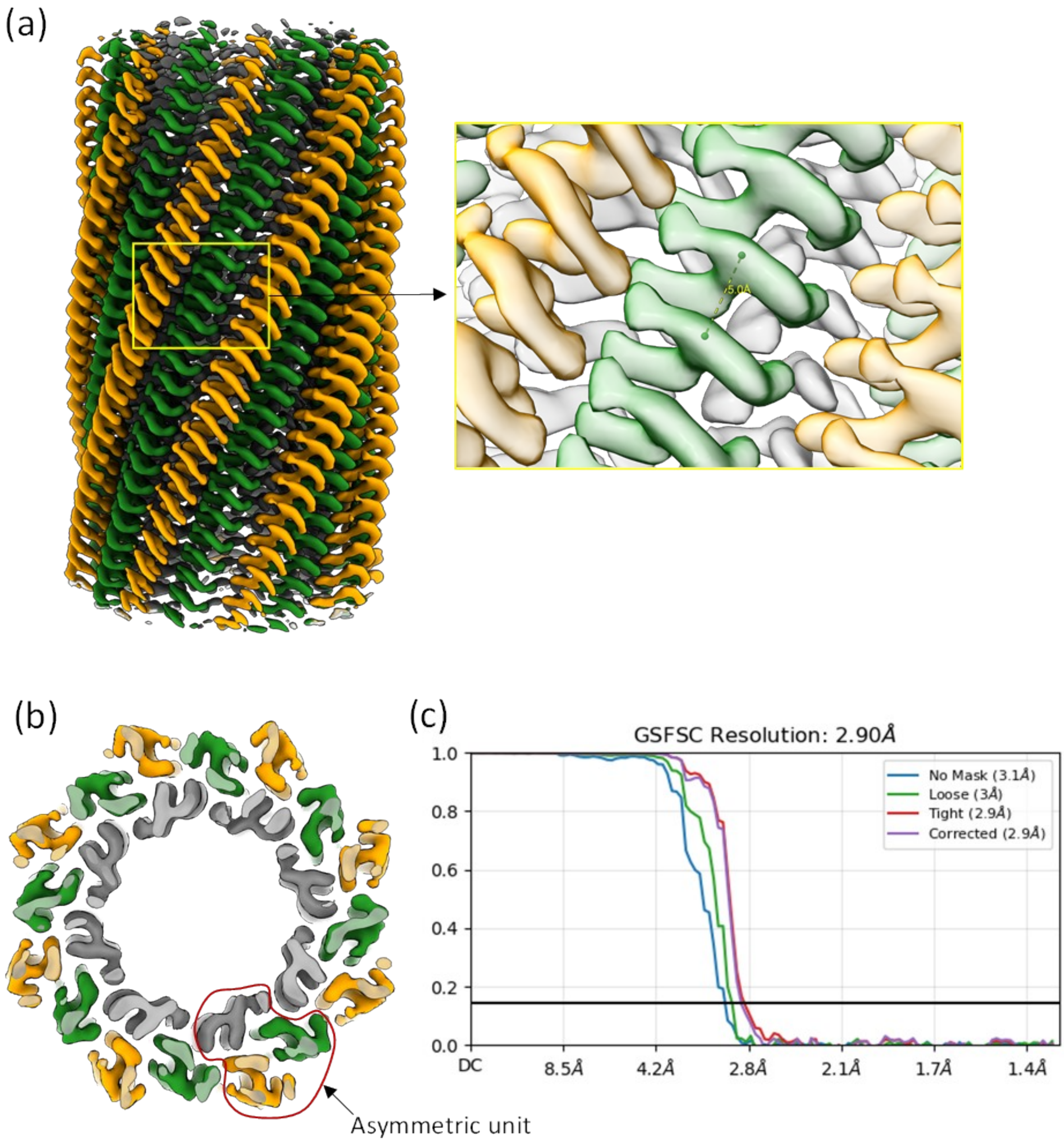


**Figure S5.** (a) Side and (b) top views of the high-resolution cryo-EM map of the tube formed by self-assembly of **D,D-2NapFF** (shown in main Figure 4b), reconstructed using helical reconstruction. The map reveals three dipeptides in the asymmetric unit and peptide stacking with an interlayer spacing of ~5 Å, consistent with the tube formed by **D,D-2NapFF** (EMDB ID: 42434). (c) Map–map Fourier shell correlation (FSC) curves (0.143 cutoff) indicating an overall map resolution of ~2.9 Å.

**Table S8.** Cryo-EM data collection and reconstruction statistics of **D,D-2NapFF** tube

| | |
|---|---|
| EMBD entry | 75070 |
| Voltage (kV) | 300 |
| Electron exposure ($e^{-}$/ Å$^{2}$) | 50 |
| Total electron dose | 50 $e^{-}$/ Å$^{2}$ |
| Magnification | 130,000x |
| Pixel size (Å/pixel) | 0.662 |
| Particle images (no.) | 287,207 |
| Helical parameters (axial rise / twist) | 0.582 Å / -134.57º |
| Resolution Map:map FSC (0.143) | 2.9 Å |

## 3. Synthesis and characterisation

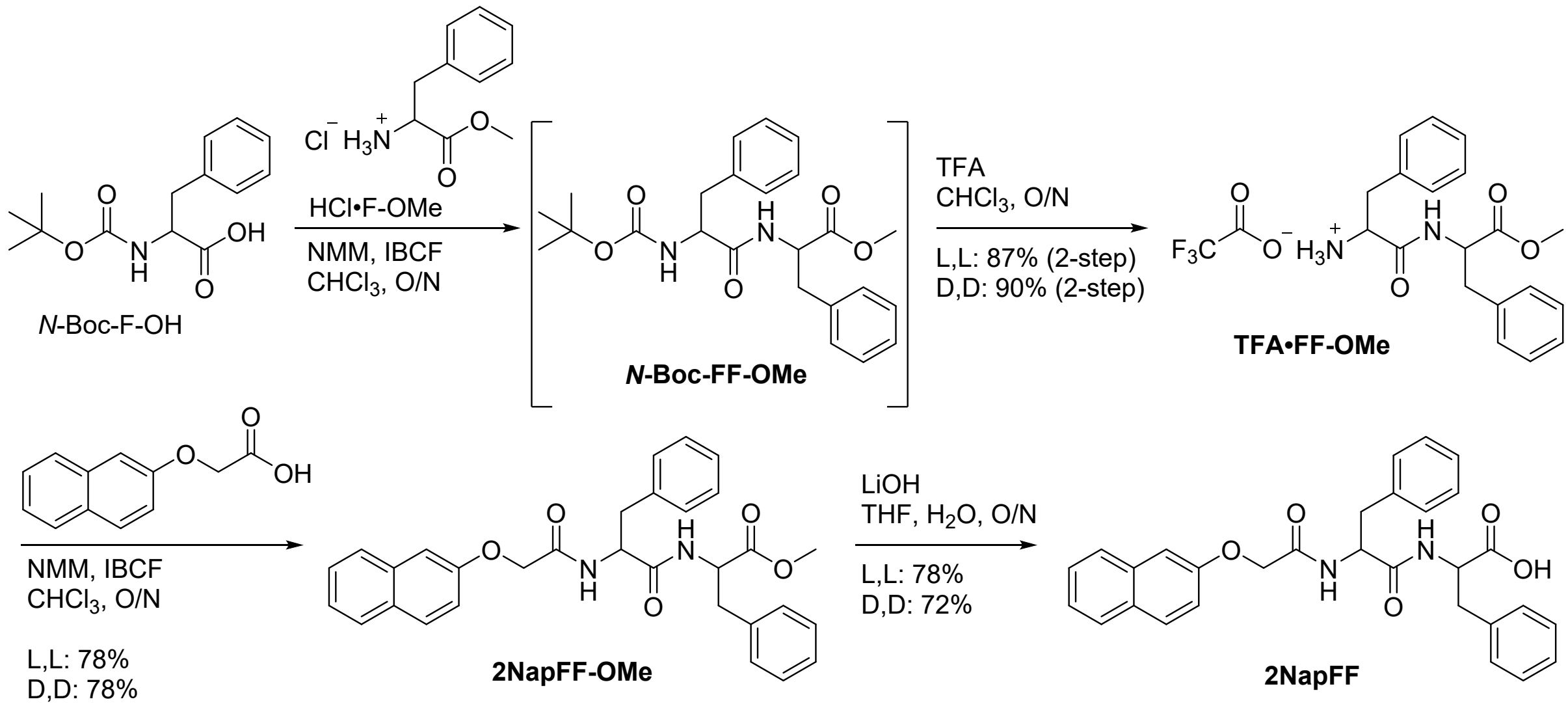


**Scheme S1**. Generalised synthetic pathway for the non-deuterated materials.

### 3.1 L,L-2NapFF

Methyl *L*-phenylalanyl-*L*-phenylalaninate trifluoroacetate salt – **TFA•L,L-FF-OMe**

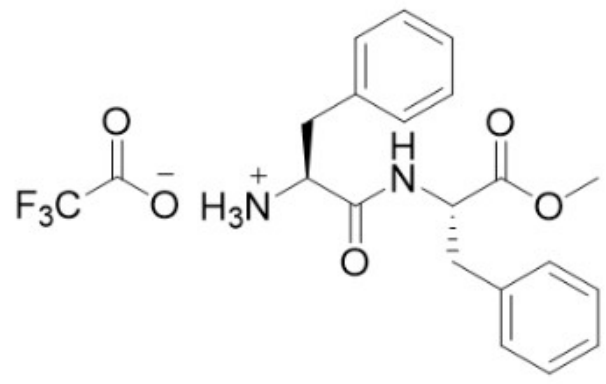

A solution of (*tert*-butoxycarbonyl)-L-phenylalanine, *N*-Boc-L-F-OH (15.0 g, 56.5 mmol) in chloroform (200 mL) was stirred and cooled in an ice/brine bath. 4-Methylmorpholine, NMM (6.23 mL, 56.7 mmol) and *iso*-butyl chloroformate, IBCF (7.40 mL, 55.3 mmol) were sequentially added during which a white precipitate formed. A sperate solution of methyl L-phenylalaninate hydrochloride, HCl•L-F-OMe (12.2 g, 56.6 mmol) and NMM (6.20 mL, 66.6 mmol) in chloroform (200 mL) was stirred until all solid dissolved and added dropwise. After addition, the resulting solution was

allowed to stir and warm to room temperature overnight. The now clear solution was diluted with hydrochloric acid (1M, 100 mL), and the layers separated. The organic layer was washed water (2 × 200 mL), dried over anhydrous sodium sulfate, and filtered on a silica plug that was washed with chloroform (*c.a.* 100 mL). The filtrate was collected, and the solvent removed to give ***N*-Boc-L,L-FF-OMe** as a white solid that was used without purification (*c.a.* 100%). ***N*-Boc-L,L-FF-OMe** was stirred in a mixture of trifluoroacetic acid (50 mL, 652 mmol) and chloroform (200 mL) overnight. The solvent and excess trifluoroacetic acid were removed to give the crude product as a viscous coloured oil. The crude oil was sonicated in diethyl ether (*c.a.* 200 mL) in which a solid precipitated. The solid was collected at the filter, washed with diethyl ether (*c.a.* 200 mL), and dried in a vacuum oven at 55 °C to give **TFA•L,L-FF-OMe** as a white solid (21.7 g, 87% 2-step) that had identical $^{1}$H NMR to that previously reported[29] and HRMS consistent with its structure; mp: 298 °C (degradation). $^{1}$H NMR (400 MHz, DMSO-d$_6$) δ: 9.10 (1H, d, *J* 7.5, N$\underline{H}$), 8.26 (3H, bs, N$\underline{H}_3$), 7.34–7.21 (10H, m, $\underline{H}_{ArF}$), 4.57 (1H, td, *J* 7.9, 6.1, C$\underline{H}_{F\mathit{F}OMe}$), 4.09 (1H, dd, *J* 7.8, 5.5, C$\underline{H}_{\mathit{F}FOMe}$), 3.60 (3H, s, C$\underline{H}_3$), 3.15–3.04 (2H, m, 1/2CH$_{2\mathit{F}\mathit{F}OMe}$), 3.01–2.94 (2H, m, 1/2CH$_{2\mathit{F}\mathit{F}OMe}$). $^{13}$C NMR (101 MHz, DMSO-d$_6$) δ: 171.2 (C=O$_{F\mathit{F}OMe}$), 168.3 (C=O$_{\mathit{F}FOMe}$), 158.2 (1C, q, $^{2}J_{C\text{-}F}$ 31.2, CF$_3$-$\underline{C}$=O), 136.7 ($\underline{C}_{ArF\mathit{F}OMe}$), 134.8 ($\underline{C}_{Ar\mathit{F}FOMe}$), 129.5 ($\underline{C}H_{ArF}$), 129.1 ($\underline{C}H_{ArF}$), 128.5 ($\underline{C}H_{ArF}$), 128.4 ($\underline{C}H_{ArF}$), 127.1 ($\underline{C}H_{ArF}$), 126.7 ($\underline{C}H_{ArF}$), 117.3 (1C, d, $^{1}J_{C\text{-}F}$ 299.6, $\underline{C}F_3$), 53.9 ($\underline{C}H_{F\mathit{F}OMe}$), 53.1 ($\underline{C}H_{\mathit{F}FOMe}$), 52.0 ($\underline{C}H_3$), 36.9 ($\underline{C}H_{2\mathit{F}FOMe}$), 36.7 ($\underline{C}H_{2F\mathit{F}OMe}$). HRMS (ESI/QTOF) *m/z*: [M+Na]$^+$ calcd for $C_{19}H_{22}N_2NaO_3$ 349.1523 (100%), 350.1554 (22%); found 349.1526 (100%), 350.1559 (22%).

Methyl [2-(naphthalen-2-yloxy)acetyl]-*L*-phenylalanyl-*L*-phenylalaninate – **L,L-2NapFF-OMe**

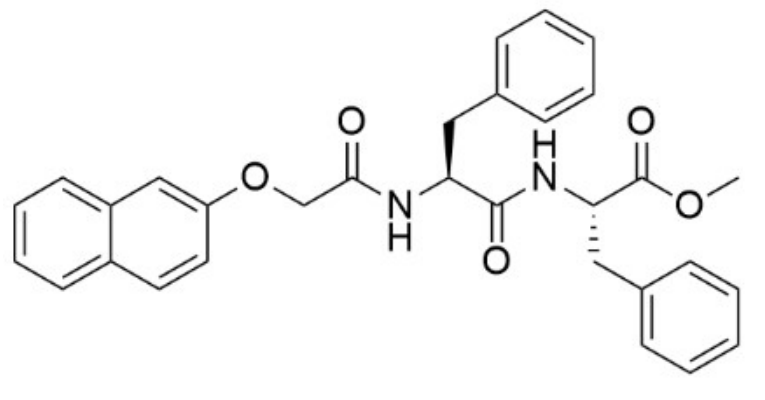

A suspension of 2-(naphthalen-2-yloxy)acetic acid (2.50 g, 12.4 mmol) and NMM (1.43 mL, 13.0 mmol) in chloroform (100 mL) was stirred until all solid was dissolved. IBCF (1.81 mL, 12.6 mmol) was added and the solution stirred. A separate solution of **TFA•L,L-FF-OMe** (5.37 g, 12.2 mmol) and NMM (1.60 mL, 14.5 mmol) in chloroform (100 mL) was stirred and sonicated until all solid dissolved and was added dropwise. After addition, the resulting solution was allowed to stir overnight. The now clear solution was diluted with hydrochloric acid (1M, 200 mL) and the layers separated. The aqueous layer was extracted with chloroform (100 mL) and the combined organic extracts washed with aqueous hydrochloric acid (1M, 100 mL), water (2 × 100 mL), brine (1 × 100 mL), dried over anhydrous sodium sulfate, and filtered. The filtrate was collected, and the solvent removed to give the crude product as a white solid. The solid was dissolved in the minimum amount of hot acetone (*c.a.* 10 mL) and poured into vigorously stirred diethyl ether (200 mL), in which a solid precipitated that was left to 1 h. The solid was collected at the filter, washed with diethyl ether (*c.a.* 100 mL), and dried in a vacuum oven at 55 °C to give **L,L-2NapFF-OMe** as a white solid (4.44 g, 78%); mp: 153 °C. $^{1}$H NMR (400 MHz, DMSO-d$_6$) δ: 8.61 (1H, d, *J* 7.5, N$\underline{H}_{F\mathit{F}OMe}$), 8.14 (1H, d, *J* 8.5, N$\underline{H}_{\mathit{F}FOMe}$), 7.86–7.81 (2H, m, $\underline{H}_{Nap}$), 7.73 (1H, d, *J* 8.3, $\underline{H}_{Nap}$), 7.46 (1H, ddd, *J* 8.2, 6.8, 1.3, $\underline{H}_{Nap}$), 7.36 (1H, ddd, *J* 8.1, 6.8, 1.2, $\underline{H}_{Nap}$), 7.27–7.12 (12H, m, $\underline{H}_{Nap}$ and $\underline{H}_{ArF}$), 4.66 (1H, td, *J* 9.1, 4.4, C$\underline{H}_{\mathit{F}FOMe}$), 4.58–

4.49 (3H, m, C<u>H</u>$_{FFOMe}$ and O-C<u>H</u>$_2$), 3.59 (3H, s, C<u>H</u>$_3$), 3.07–2.92 (3H, m, 1/2CH$_{2FFOMe}$ and CH$_{2FFOMe}$), 2.85 (1H, dd, *J* 13.8, 9.5, 1/2CH$_{2FFOMe}$). $^{13}$C NMR (101 MHz, DMSO-d$_6$) δ: 171.7 (<u>C</u>=O$_{FFOMe}$), 170.9 (<u>C</u>=O$_{FFOMe}$), 167.2 (CH$_2$-<u>C</u>=O), 155.5 (<u>C</u>$_{Nap}$-O), 137.4 (<u>C</u>$_{ArFFOMe}$), 136.9 (<u>C</u>$_{ArFFOMe}$), 134.0 (<u>C</u>$_{Nap}$), 129.3 (<u>C</u>H$_{Nap}$), 129.2 (<u>C</u>H$_{ArF}$), 129.0 (<u>C</u>H$_{ArF}$), 128.7 (<u>C</u>$_{Nap}$), 128.2 (<u>C</u>H$_{ArF}$), 128.0 (<u>C</u>H$_{ArF}$), 127.5 (<u>C</u>H$_{Nap}$), 126.7 (<u>C</u>H$_{Nap}$), 126.5 (<u>C</u>H$_{ArF}$), 126.4 (<u>C</u>H$_{Nap}$), 126.2 (<u>C</u>H$_{ArF}$), 123.8 (<u>C</u>H$_{Nap}$), 118.4 (<u>C</u>H$_{Nap}$), 107.3 (<u>C</u>H$_{Nap}$), 66.6 (O-<u>C</u>H$_2$), 53.6 (<u>C</u>H$_{FFOMe}$), 53.1 (<u>C</u>H$_{FFOMe}$), 51.8 (<u>C</u>H$_3$), 37.4 (<u>C</u>H$_{2FFOMe}$), 36.6 (<u>C</u>H$_{2FFOMe}$). HRMS (ESI/QTOF) *m/z*: [M+Na]$^+$ calcd for $C_{31}H_{30}N_2NaO_5$ 533.2047 (100%), 534.2079 (35%); found 533.2044 (100%), 5340.2077 (34%).

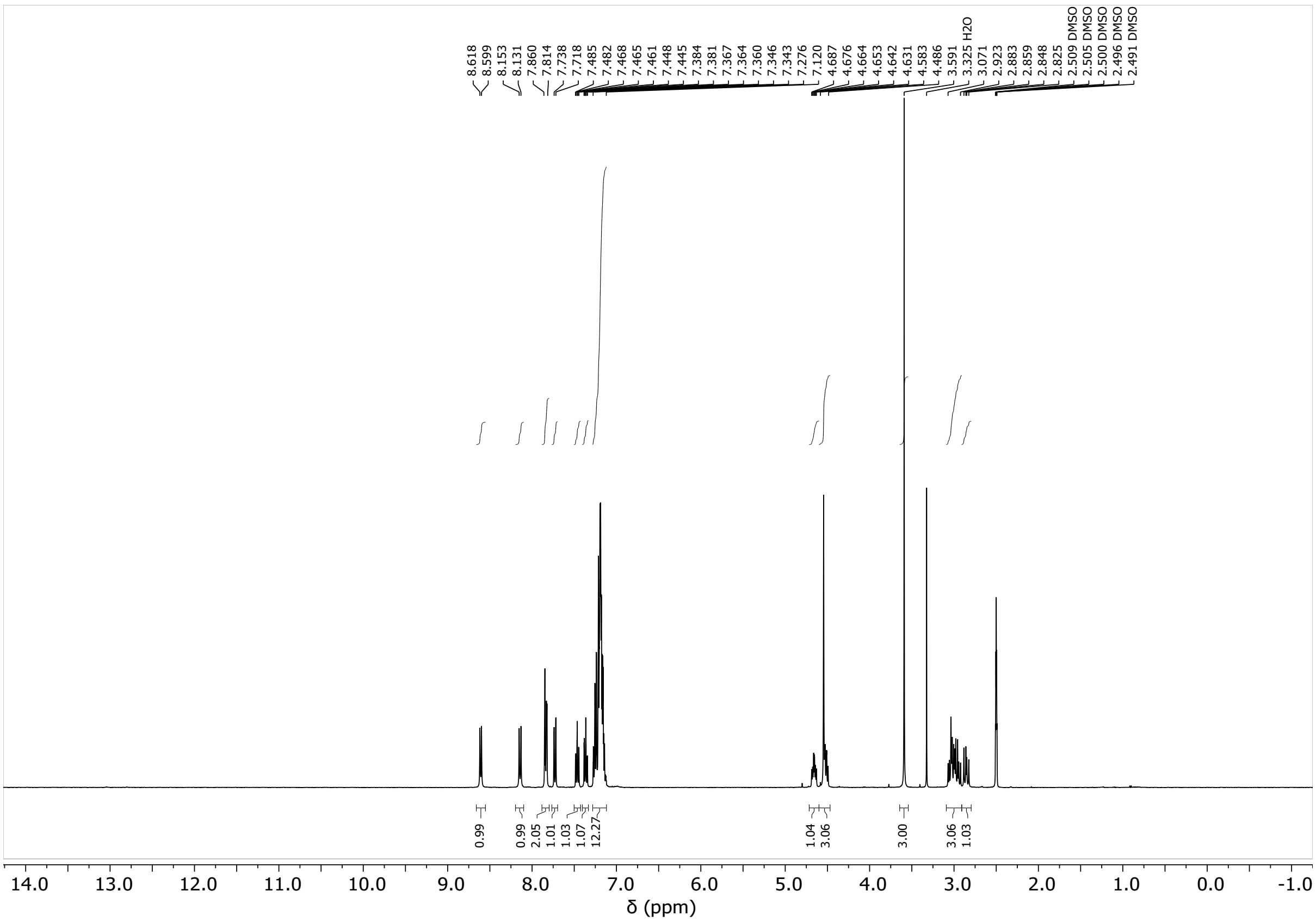

**Figure S6**. $^1$H NMR spectrum (DMSO-d$_6$, 400 MHz) of **L,L-2NapFF-OMe**.

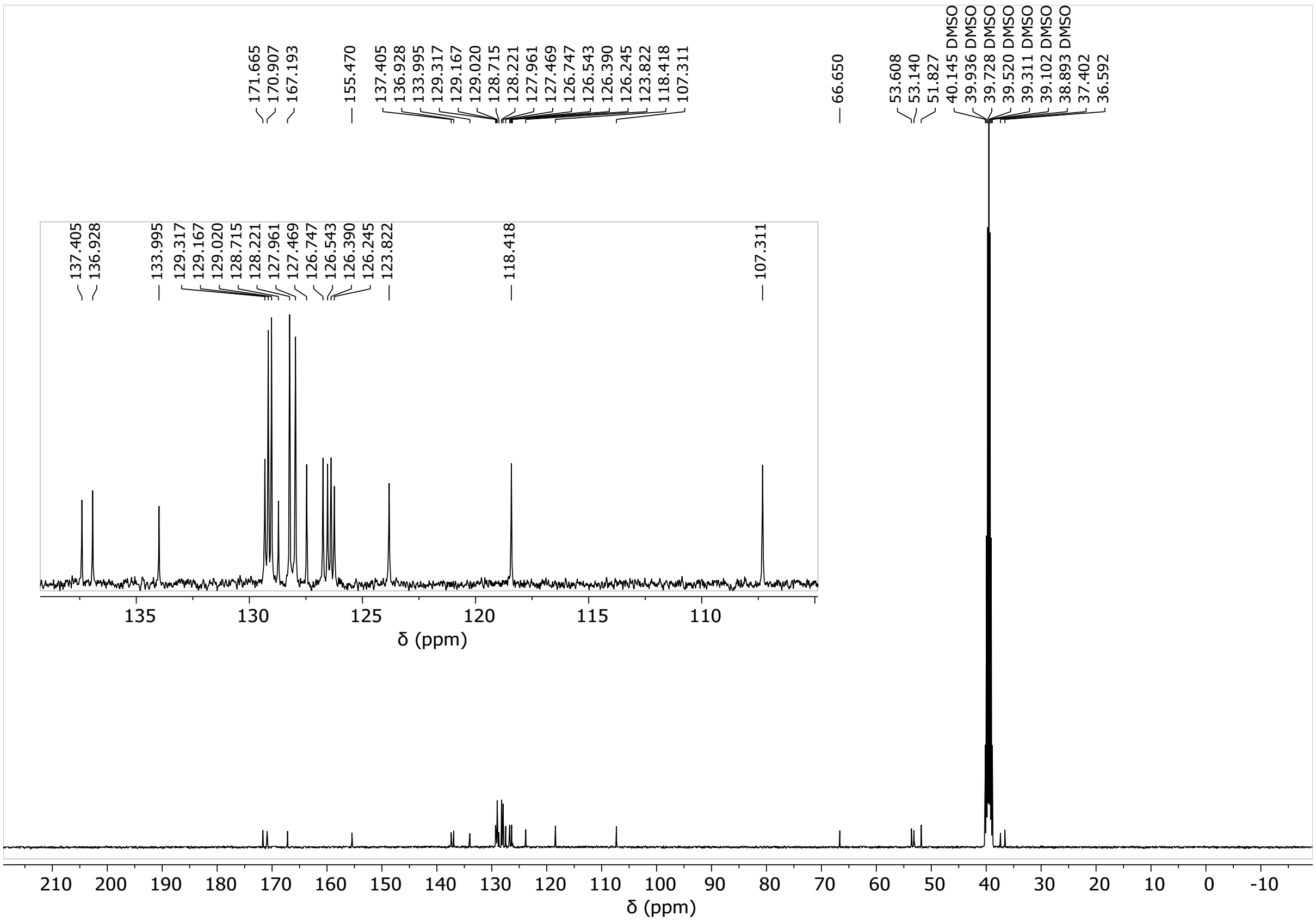


**Figure S7**. $^{13}$C NMR spectrum (DMSO-d$_6$, 101 MHz) of **L,L-2NapFF-OMe**.

[2-(Naphthalen-2-yloxy)acetyl]-*L*-phenylalanyl-*L*-phenylalanine – **L,L-2NapFF**.

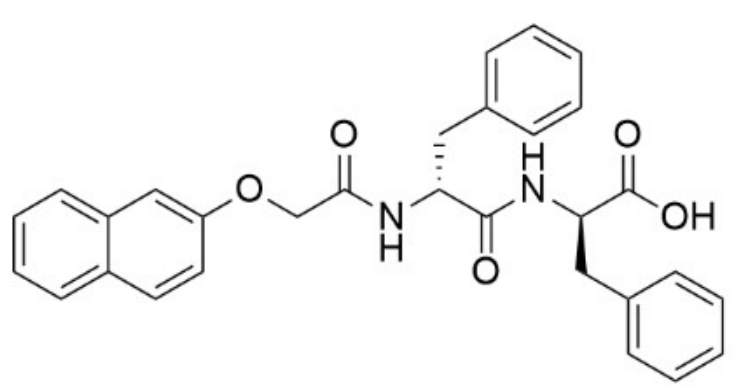


A suspension of **L,L-2NapFF-OMe** (4.30 g, 8.42 mmol) and lithium hydroxide (0.81 g, 33.7 mmol) in tetrahydrofuran (50 mL) and water (50 mL) was stirred overnight. The now clear solution was slowly poured into vigorously stirred aqueous hydrochloric acid (1M, 100 mL) to give a white precipitate. The suspension was allowed to stir for 1 h, and the solid was collected at the filter and washed with water (3 × 100 mL). The solid was sonicated in an acetonitrile:diethyl ether mixture (1:1; 100 mL) for 1 h, collected at the filter. The crude solid was then recrystalised from boiling acetonitrile, collected at the filter, and dried in a vacuum oven at 55 °C to give **L,L-2NapFF** as a white solid (3.26 g, 78%); mp: 159 °C. $^1$H NMR (400 MHz, DMSO-d$_6$) δ: 12.83 (1H, s, COO$\underline{H}$), 8.45 (1H, d, *J* 7.8, N$\underline{H}_{\text{F}\textit{F}\text{OH}}$), 8.13 (1H, d, *J* 8.6, N$\underline{H}_{\textit{F}\text{FOH}}$), 7.85–7.82 (2H, m, $\underline{H}_{\text{Nap}}$), 7.73 (1H, d, *J* 8.2, $\underline{H}_{\text{Nap}}$), 7.47 (1H, ddd, *J* 8.2, 6.8, 1.3, $\underline{H}_{\text{Nap}}$), 7.36 (1H, ddd, *J* 8.2, 6.9, 1.3, $\underline{H}_{\text{Nap}}$), 7.28–7.11 (12H, m, $\underline{H}_{\text{Nap}}$ and $\underline{H}_{\text{ArF}}$), 4.67 (1H, td, *J* 9.4, 4.2, C$\underline{H}_{\textit{F}\text{FOH}}$), 4.55 (2H, s, O-C$\underline{H}_2$), 4.50 (1H, td, *J* 8.6, 5.4, C$\underline{H}_{\text{F}\textit{F}\text{OH}}$), 3.07 (2H, ddd, *J* 18.9, 13.9, 4.8, 1/2C$\underline{H}_{2\text{F}}$), 2.97–2.84 (2H, m, 1/2C$\underline{H}_{2\text{F}}$). $^{13}$C NMR (101 MHz, DMSO-d$_6$) δ: 172.7 ($\underline{C}$OOH), 170.8 ($\underline{C}$=O$_{\textit{F}\text{FOH}}$), 167.2 (CH$_2$-$\underline{C}$=O), 155.5 ($\underline{C}_{\text{Nap}}$-O), 137.5 ($\underline{C}_{\text{Ar}\textit{F}\text{FOH}}$), 137.3 ($\underline{C}_{\text{ArF}\textit{F}\text{OH}}$), 134.0 ($\underline{C}_{\text{Nap}}$), 129.3 ($\underline{C}$H$_{\text{Nap}}$), 129.2 ($\underline{C}$H$_{\text{ArF}}$), 129.1 ($\underline{C}$H$_{\text{ArF}}$), 128.7 ($\underline{C}_{\text{Nap}}$), 128.2 ($\underline{C}$H$_{\text{ArF}}$), 127.9 ($\underline{C}$H$_{\text{ArF}}$), 127.5 ($\underline{C}$H$_{\text{Nap}}$), 126.8 ($\underline{C}$H$_{\text{Nap}}$), 126.4 ($\underline{C}$H$_{\text{Nap}}$ and $\underline{C}$H$_{\text{ArF}}$), 126.2 ($\underline{C}$H$_{\text{ArF}}$), 123.8 ($\underline{C}$H$_{\text{Nap}}$), 118.4 ($\underline{C}$H$_{\text{Nap}}$), 107.3 ($\underline{C}$H$_{\text{Nap}}$), 66.7 (O-$\underline{C}$H$_2$), 53.5 ($\underline{C}$H$_{\text{F}\textit{F}\text{OH}}$), 53.2 ($\underline{C}$H$_{\textit{F}\text{FOH}}$), 37.4 ($\underline{C}$H$_{2\textit{F}\text{FOH}}$), 36.7 ($\underline{C}$H$_{2\text{F}\textit{F}\text{OH}}$). HRMS (ESI/QTOF) *m/z*: [M+Na]$^+$ calcd for $C_{30}H_{28}N_2NaO_5$ 519.1890 (100%), 520.1922 (34%); found 519.1894 (100%), 520.1926 (35%).

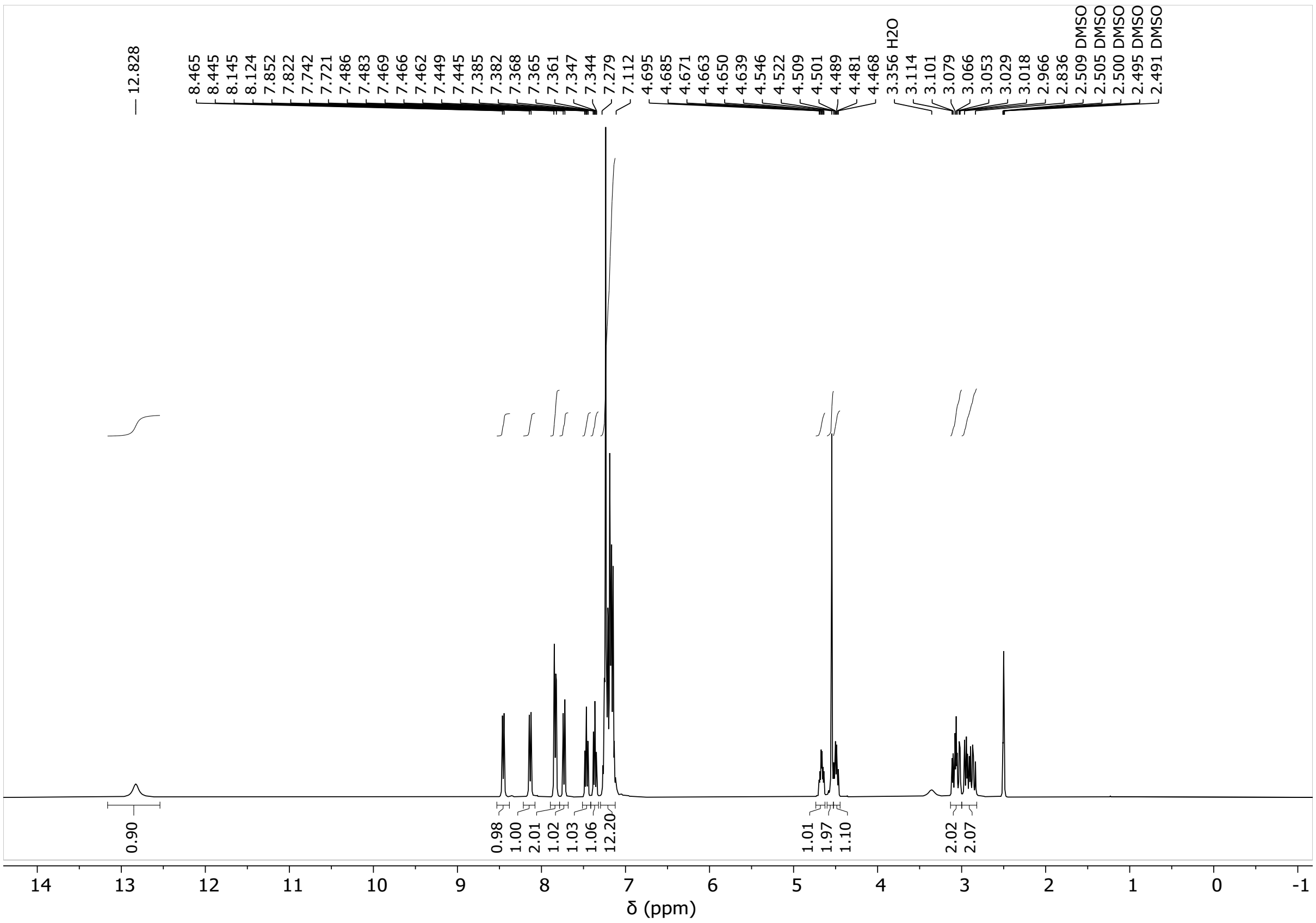

Figure S8. $^{1}H$ NMR spectrum (DMSO-$d_6$, 400 MHz) of **L,L-2NapFF**.

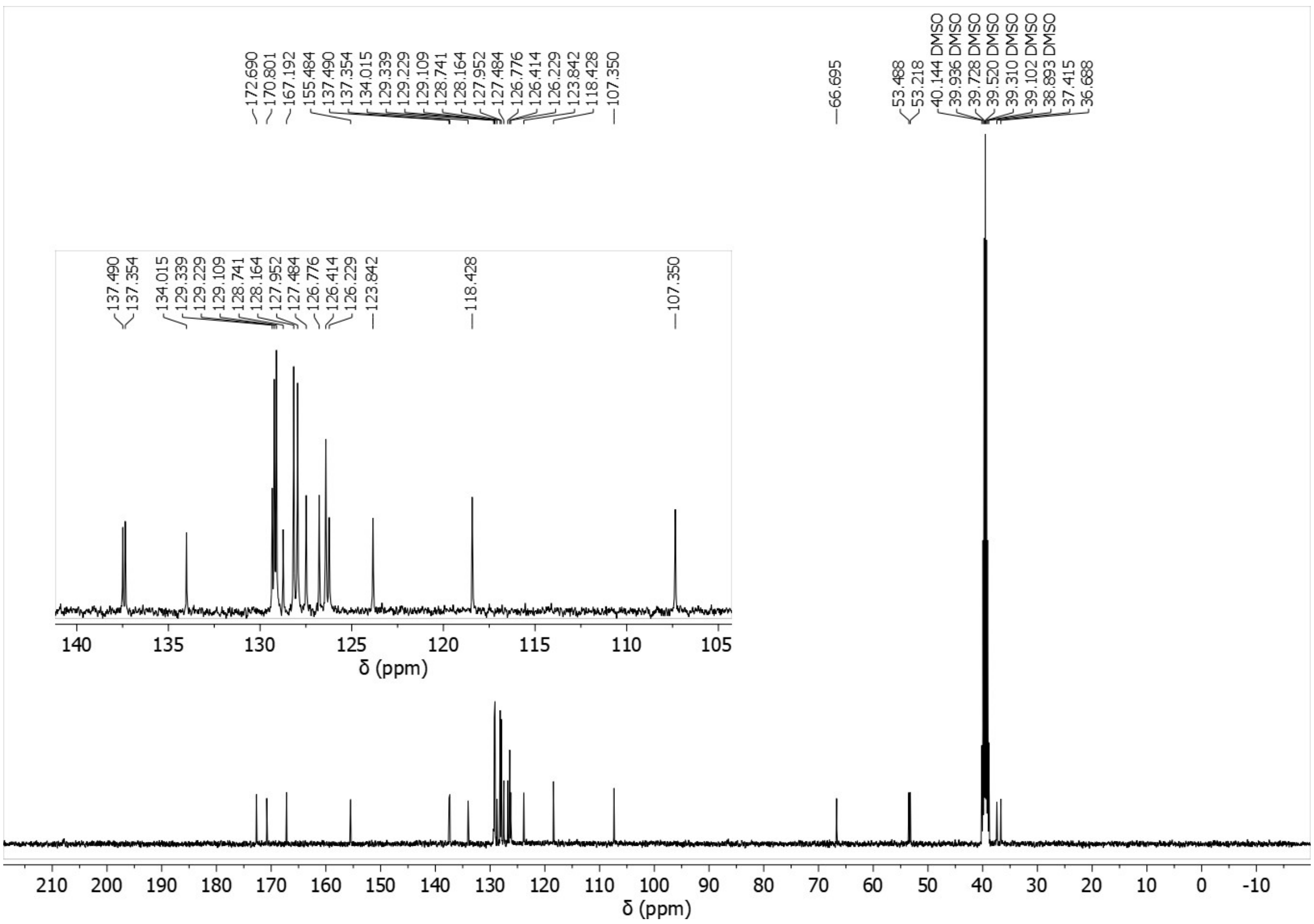

**Figure S9.** $^{13}$C NMR spectrum (DMSO-d$_6$, 101 MHz) of **L,L-2NapFF**.

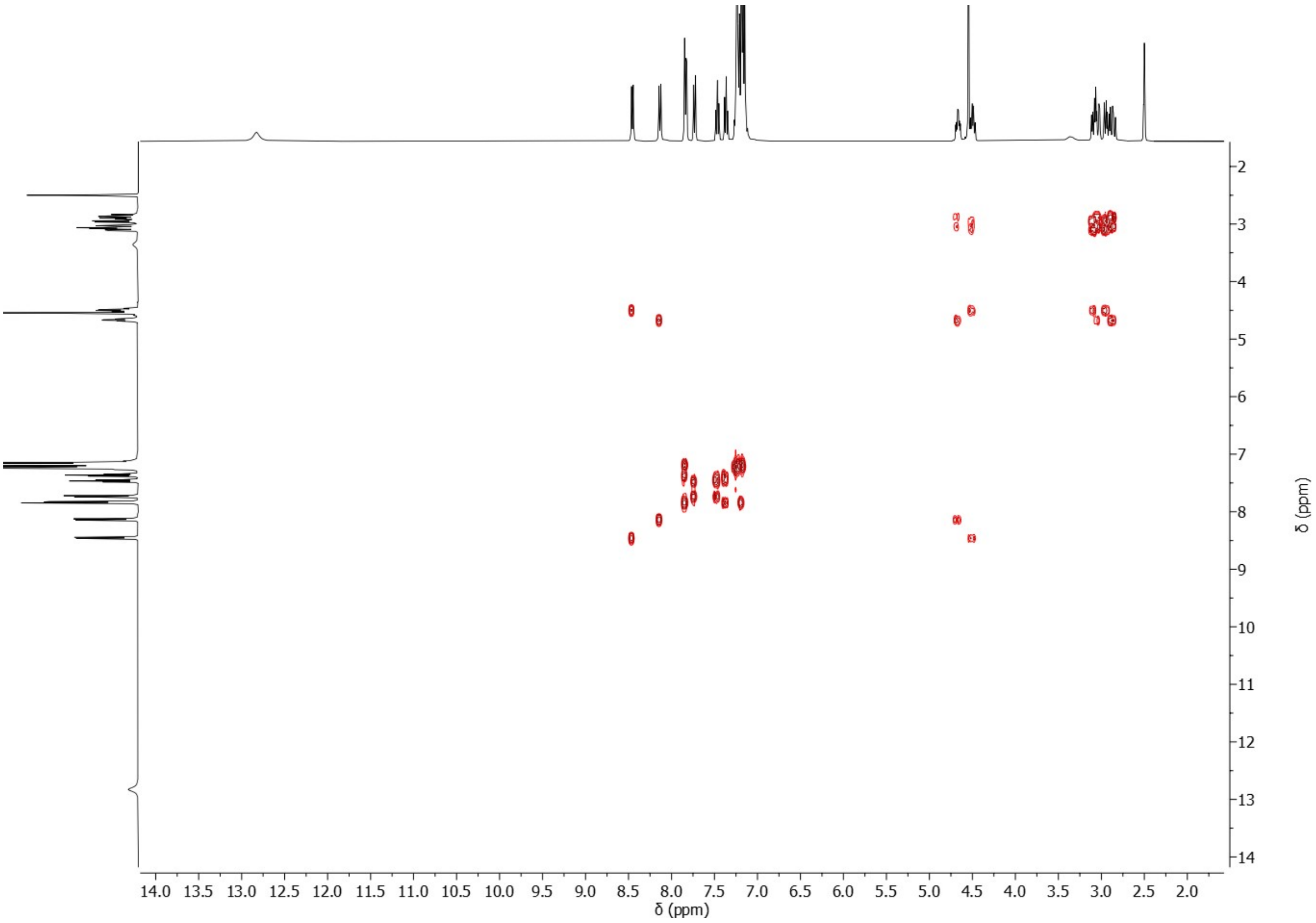


**Figure S10**. COSY NMR (DMSO-d$_6$) of **L,L-2NapFF**.

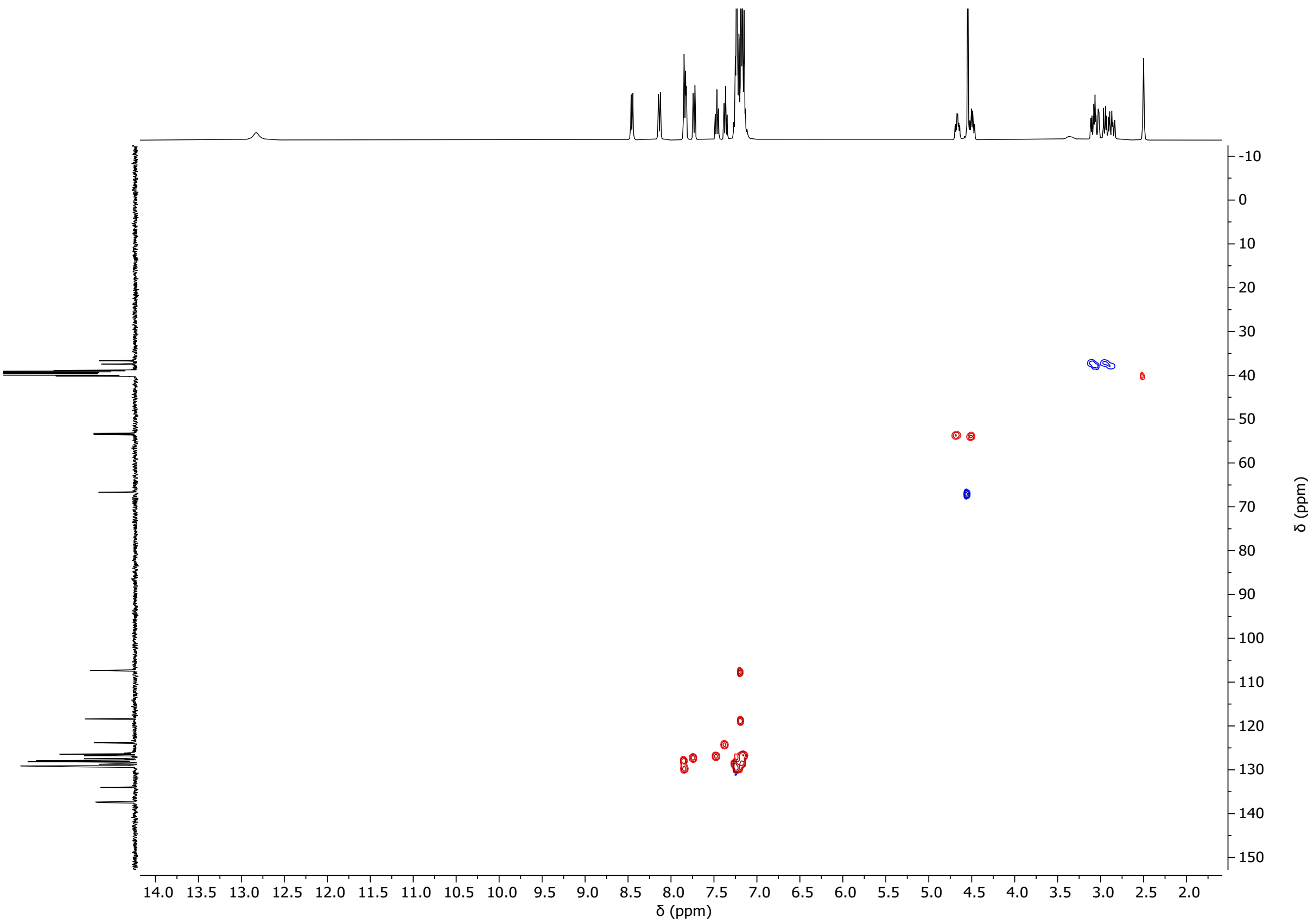

**Figure S11**. HSQC NMR (DMSO-$d_6$) of **L,L-2NapFF**.

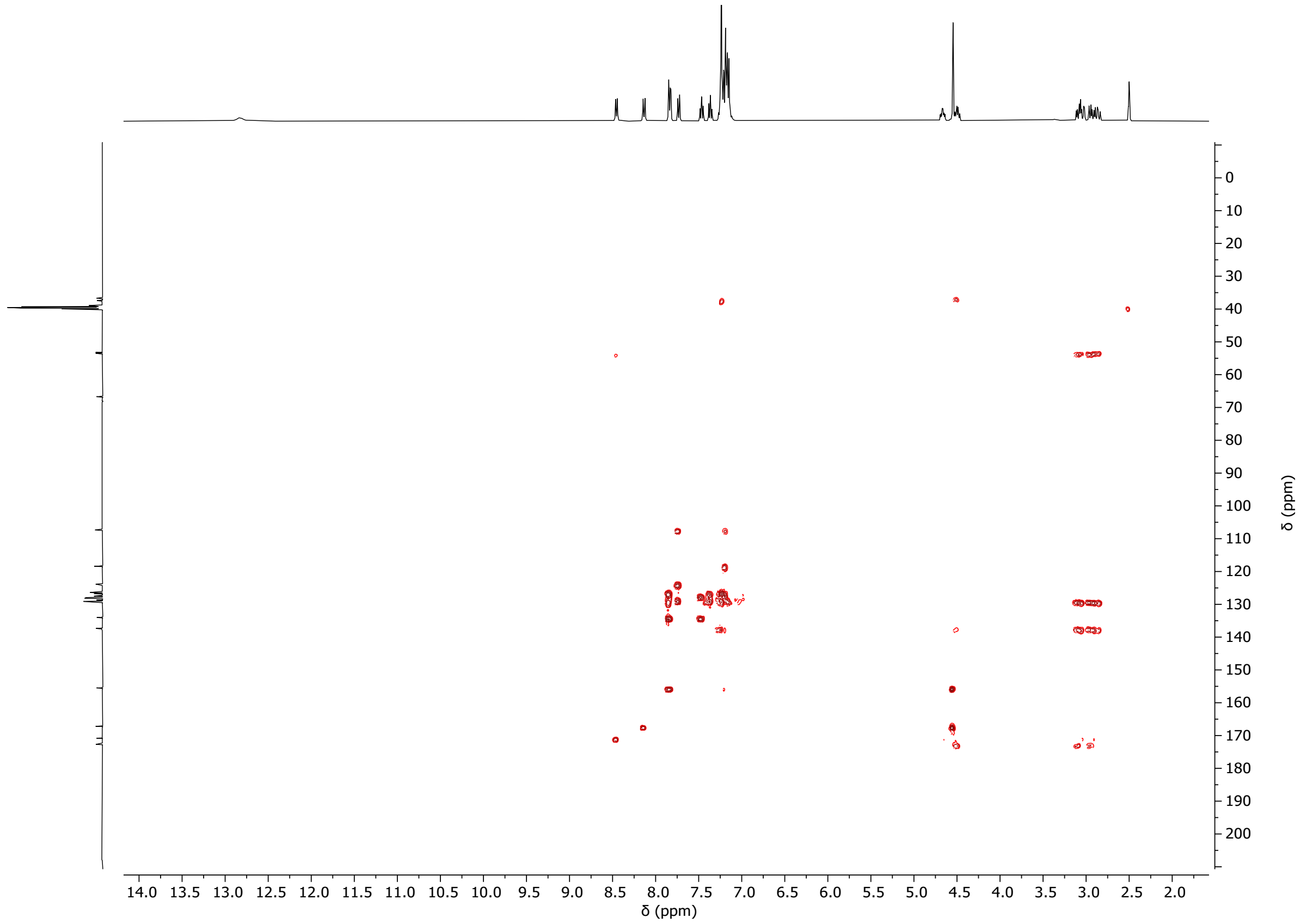


**Figure S12**. HMBC NMR (DMSO-$d_6$) of **L,L-2NapFF**.

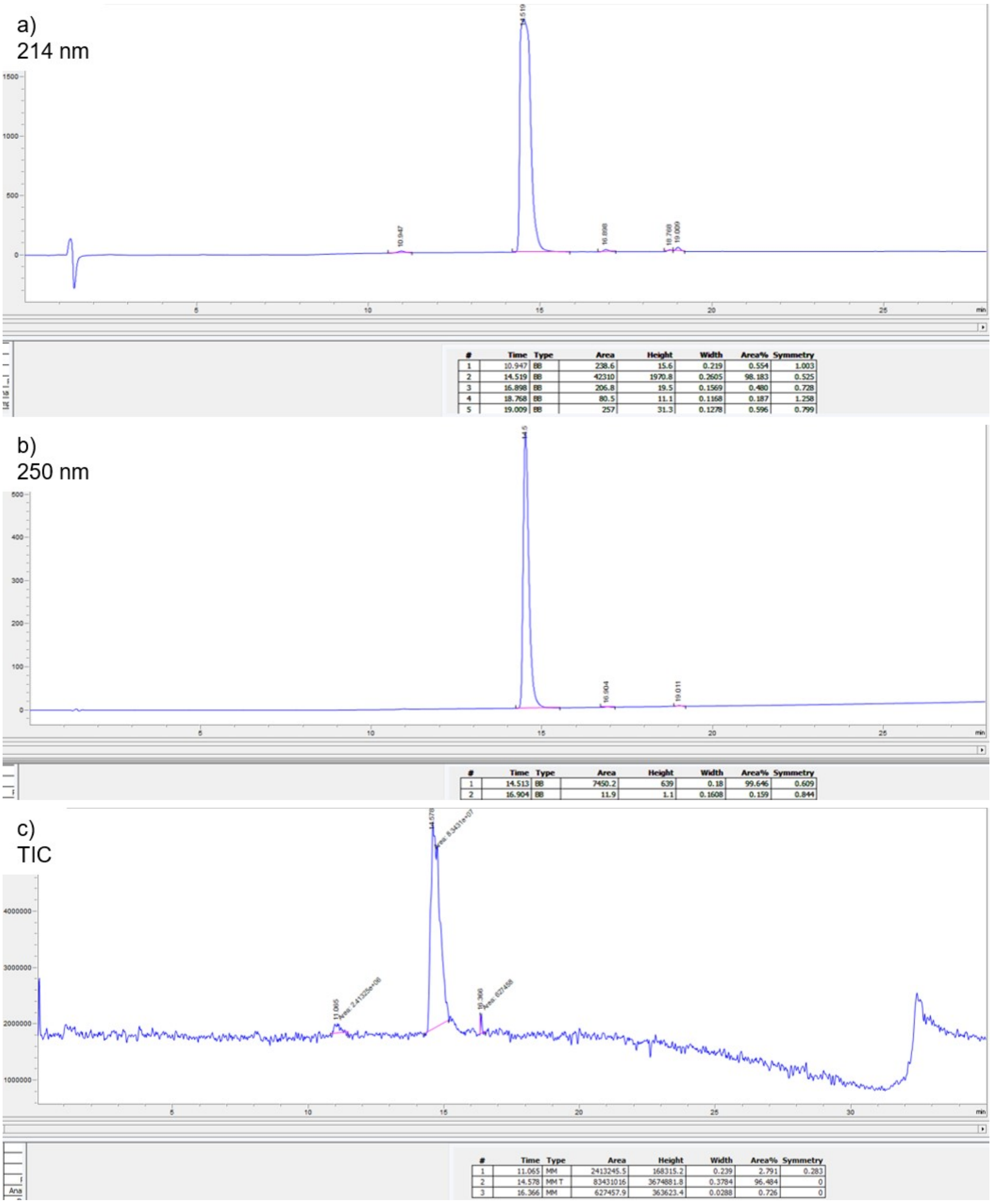


| # | Time | Type | Area | Height | Width | Area% | Symmetry |
|---|---|---|---|---|---|---|---|
| 1 | 10.947 | BB | 238.6 | 15.6 | 0.219 | 0.554 | 1.003 |
| 2 | 14.519 | BB | 42310 | 1970.8 | 0.2605 | 98.183 | 0.525 |
| 3 | 16.898 | BB | 206.8 | 19.5 | 0.1569 | 0.480 | 0.728 |
| 4 | 18.768 | BB | 80.5 | 11.1 | 0.1168 | 0.187 | 1.258 |
| 5 | 19.009 | BB | 257 | 31.3 | 0.1278 | 0.596 | 0.799 |

| # | Time | Type | Area | Height | Width | Area% | Symmetry |
|---|---|---|---|---|---|---|---|
| 1 | 14.513 | BB | 7450.2 | 639 | 0.18 | 99.646 | 0.609 |
| 2 | 16.904 | BB | 11.9 | 1.1 | 0.1608 | 0.159 | 0.844 |

| # | Time | Type | Area | Height | Width | Area% | Symmetry |
|---|---|---|---|---|---|---|---|
| 1 | 11.065 | MM | 2413245.5 | 168315.2 | 0.239 | 2.791 | 0.283 |
| 2 | 14.578 | MM T | 83431016 | 3674881.8 | 0.3784 | 96.484 | 0 |
| 3 | 16.366 | MM | 627457.9 | 363623.4 | 0.0288 | 0.726 | 0 |

**Figure S13**. HPLC-ESI-MS chromatograms of **L,L-2NapFF** from the a) 214 nm and b) 250 nm detector, and c) total ion chromatogram (TIC). Conditions: C18 reversed-phase column; linear gradient from 40% to 90% acetonitrile in water over 20 min; flow rate 0.30 mL min$^{-1}$; injection volume 5 µL; sample concentration 2 mg mL$^{-1}$; room temperature.

**Compound Details**

**Cpd. 1: C30 H28 N2 O5**

| Name | Formula | Mass (Tgt) | Mass | Diff (Tgt, ppm) | Score (Tgt) |
|---|---|---|---|---|---|
| | C30 H28 N2 O5 | 496.199822 | 496.200057 | 0.47 | 99.71 |

*Compound Spectra (overlaid)*

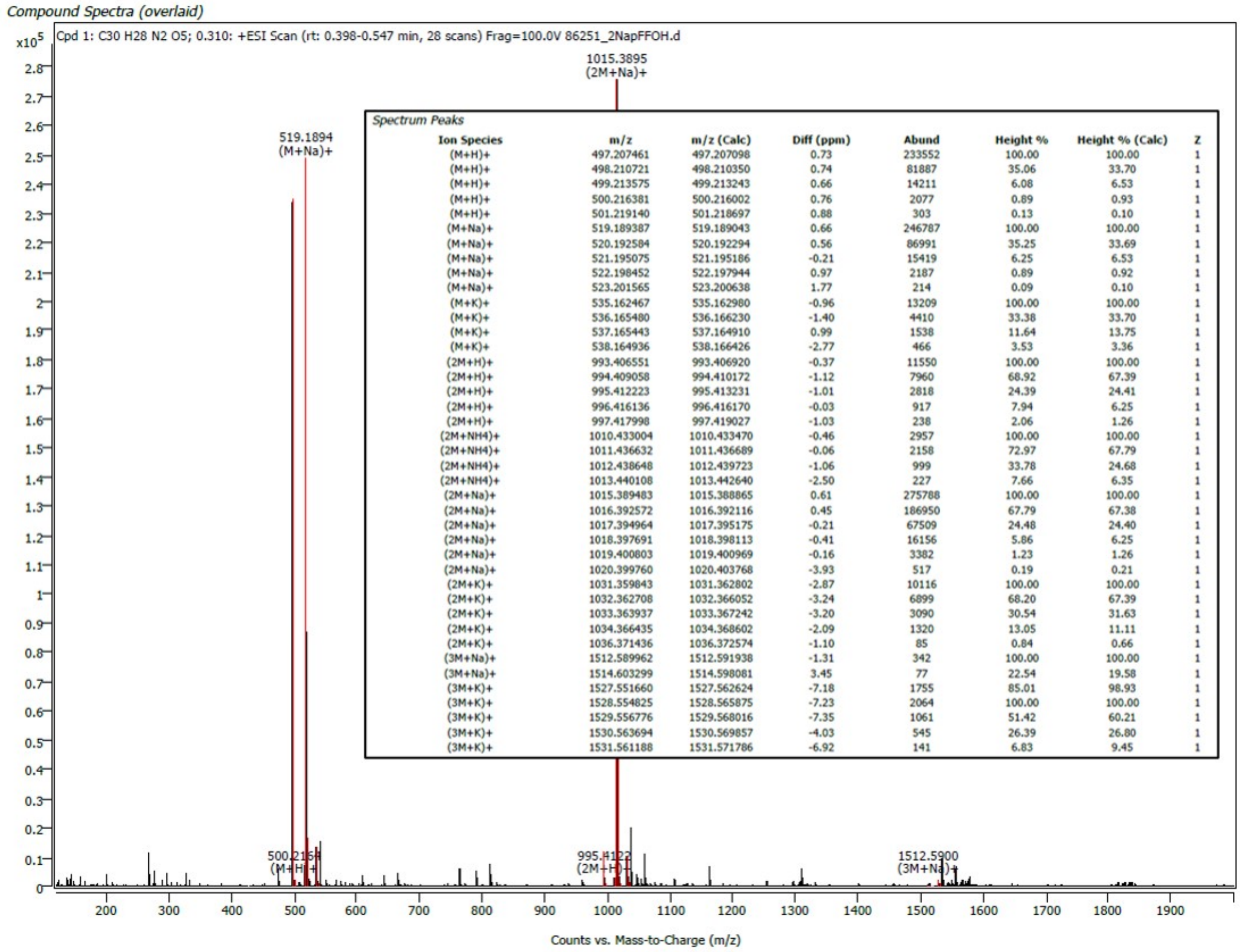


| Ion Species | m/z | m/z (Calc) | Diff (ppm) | Abund | Height % | Height % (Calc) | Z |
|---|---|---|---|---|---|---|---|
| (M+H)+ | 497.207461 | 497.207098 | 0.73 | 233552 | 100.00 | 100.00 | 1 |
| (M+H)+ | 498.210721 | 498.210350 | 0.74 | 81887 | 35.06 | 33.70 | 1 |
| (M+H)+ | 499.213575 | 499.213243 | 0.66 | 14211 | 6.08 | 6.53 | 1 |
| (M+H)+ | 500.216381 | 500.216002 | 0.76 | 2077 | 0.89 | 0.93 | 1 |
| (M+H)+ | 501.219140 | 501.218697 | 0.88 | 303 | 0.13 | 0.10 | 1 |
| (M+Na)+ | 519.189387 | 519.189043 | 0.66 | 246787 | 100.00 | 100.00 | 1 |
| (M+Na)+ | 520.192584 | 520.192294 | 0.56 | 86991 | 35.25 | 33.69 | 1 |
| (M+Na)+ | 521.195075 | 521.195186 | -0.21 | 15419 | 6.25 | 6.53 | 1 |
| (M+Na)+ | 522.198452 | 522.197944 | 0.97 | 2187 | 0.89 | 0.92 | 1 |
| (M+Na)+ | 523.201565 | 523.200638 | 1.77 | 214 | 0.09 | 0.10 | 1 |
| (M+K)+ | 535.162467 | 535.162980 | -0.96 | 13209 | 100.00 | 100.00 | 1 |
| (M+K)+ | 536.165480 | 536.166230 | -1.40 | 4410 | 33.38 | 33.70 | 1 |
| (M+K)+ | 537.165443 | 537.164910 | 0.99 | 1538 | 11.64 | 13.75 | 1 |
| (M+K)+ | 538.164936 | 538.166426 | -2.77 | 466 | 3.53 | 3.36 | 1 |
| (2M+H)+ | 993.406551 | 993.406920 | -0.37 | 11550 | 100.00 | 100.00 | 1 |
| (2M+H)+ | 994.409058 | 994.410172 | -1.12 | 7960 | 68.92 | 67.39 | 1 |
| (2M+H)+ | 995.412223 | 995.413231 | -1.01 | 2818 | 24.39 | 24.41 | 1 |
| (2M+H)+ | 996.416136 | 996.416170 | -0.03 | 917 | 7.94 | 6.25 | 1 |
| (2M+H)+ | 997.417998 | 997.419027 | -1.03 | 238 | 2.06 | 1.26 | 1 |
| (2M+NH4)+ | 1010.433004 | 1010.433470 | -0.46 | 2957 | 100.00 | 100.00 | 1 |
| (2M+NH4)+ | 1011.436632 | 1011.436689 | -0.06 | 2158 | 72.97 | 67.79 | 1 |
| (2M+NH4)+ | 1012.438648 | 1012.439723 | -1.06 | 999 | 33.78 | 24.68 | 1 |
| (2M+NH4)+ | 1013.440108 | 1013.442640 | -2.50 | 227 | 7.66 | 6.35 | 1 |
| (2M+Na)+ | 1015.389483 | 1015.388865 | 0.61 | 275788 | 100.00 | 100.00 | 1 |
| (2M+Na)+ | 1016.392572 | 1016.392116 | 0.45 | 186950 | 67.79 | 67.38 | 1 |
| (2M+Na)+ | 1017.394964 | 1017.395175 | -0.21 | 67509 | 24.48 | 24.40 | 1 |
| (2M+Na)+ | 1018.397691 | 1018.398113 | -0.41 | 16156 | 5.86 | 6.25 | 1 |
| (2M+Na)+ | 1019.400803 | 1019.400969 | -0.16 | 3382 | 1.23 | 1.26 | 1 |
| (2M+Na)+ | 1020.399760 | 1020.403768 | -3.93 | 517 | 0.19 | 0.21 | 1 |
| (2M+K)+ | 1031.359843 | 1031.362802 | -2.87 | 10116 | 100.00 | 100.00 | 1 |
| (2M+K)+ | 1032.362708 | 1032.366052 | -3.24 | 6899 | 68.20 | 67.39 | 1 |
| (2M+K)+ | 1033.363937 | 1033.367242 | -3.20 | 3090 | 30.54 | 31.63 | 1 |
| (2M+K)+ | 1034.366435 | 1034.368602 | -2.09 | 1320 | 13.05 | 11.11 | 1 |
| (2M+K)+ | 1036.371436 | 1036.372574 | -1.10 | 85 | 0.84 | 0.66 | 1 |
| (3M+Na)+ | 1512.589962 | 1512.591938 | -1.31 | 342 | 100.00 | 100.00 | 1 |
| (3M+Na)+ | 1514.603299 | 1514.598081 | 3.45 | 77 | 22.54 | 19.58 | 1 |
| (3M+K)+ | 1527.551660 | 1527.562624 | -7.18 | 1755 | 85.01 | 98.93 | 1 |
| (3M+K)+ | 1528.554825 | 1528.565875 | -7.23 | 2064 | 100.00 | 100.00 | 1 |
| (3M+K)+ | 1529.556776 | 1529.568016 | -7.35 | 1061 | 51.42 | 60.21 | 1 |
| (3M+K)+ | 1530.563694 | 1530.569857 | -4.03 | 545 | 26.39 | 26.80 | 1 |
| (3M+K)+ | 1531.561188 | 1531.571786 | -6.92 | 141 | 6.83 | 9.45 | 1 |

**Figure S14**. ESI-HRMS spectrum of **L,L-2NapFF** with peak table inset.

### 3.2 D,D-2NapFF

Methyl *D*-phenylalanyl-*D*-phenylalaninate trifluoroacetate salt – **TFA•D,D-FF-OMe**

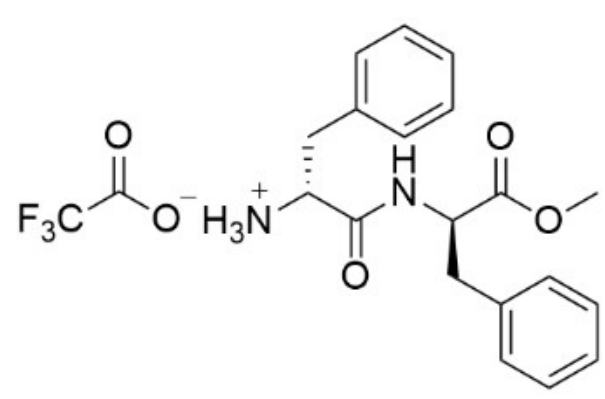

A solution of (*tert*-butoxycarbonyl)-D-phenylalanine, *N*-Boc-D-F-OH (11.5 g, 43.3 mmol) in chloroform (200 mL) was stirred and cooled in an ice/brine bath for 20 min. 4-Methylmorpholine, NMM (5.24 mL, 47.7 mmol) and *iso*-butyl chloroformate, IBCF (5.60 mL, 43.3 mmol) were sequentially added, and the solution left to stir for 20 min in the cold bath during which a white precipitate formed. A sperate solution of methyl D-phenylalaninate hydrochloride, HCl•*D*-F-OMe (9.35 g, 43.3 mmol) and NMM (5.5 mL, 50.0 mmol) in chloroform (200 mL) was stirred until all solid dissolved and added dropwise. After addition, the resulting solution was allowed to stir and warm to room temperature overnight. The now clear solution was diluted with hydrochloric acid (1M, 200 mL), stirred for 20 min, and the layers separated. The aqueous layer was extracted with chloroform (100 mL) and the combined organic extracts washed with hydrochloric acid (1M, 200 mL), water (2 × 200 mL), brine (200 mL), dried over anhydrous sodium sulfate, and filtered on a silica plug

that was washed with chloroform (100 mL). The filtrate was collected, and the solvent removed to give ***N*-Boc-D,D-FF-OMe** as a white solid that was used without purification (*c.a.* 100%). ***N*-Boc-D,D-FF-OMe** was stirred in a mixture of trifluoroacetic acid (200 mL, 2.61 mol) and chloroform (250 mL) overnight. The solvent and excess trifluoroacetic acid were removed to give the crude product as a viscous coloured oil that solidified upon standing. The crude solid was dissolved in chloroform (50 mL) with sonication and slowed poured into vigorously stirred diethyl ether (400 mL) in which a solid precipitated that was allowed to stir overnight. The solid was collected at the filter, washed with diethyl ether (200 mL), and dried in an oven at 100 °C to give **TFA•D,D-FF-OMe** as a white solid (17.1 g, 90% 2-step) that had identical HRMS to that previously reported[30] and NMR consistent with its structure; mp: 286 °C (degradation). $^{1}$H NMR (400 MHz, DMSO-d$_6$) δ: 9.00 (1H, d, *J* 7.7, $N\underline{H}$), 8.14 (3H, bs, $N\underline{H}_3$), 7.36–7.21 (10H, m, $\underline{H}_{ArF}$), 4.58 (1H, td, *J* 7.9, 6.2, $C\underline{H}_{F\mathit{F}OMe}$), 4.04 (1H, dd, *J* 8.2, 5.3, $C\underline{H}_{\mathit{F}FOMe}$), 3.61 (3H, s, $C\underline{H}_3$), 3.13–3.04 (2H, m, 1/2$CH_{2\mathit{F}\mathit{F}OMe}$), 3.01–2.89 (2H, m, 1/2$CH_{2\mathit{F}\mathit{F}OMe}$). $^{13}$C NMR (101 MHz, DMSO-d$_6$) δ: 171.1 ($C{=}O_{F\mathit{F}OMe}$), 168.2 ($C{=}O_{\mathit{F}FOMe}$), 157.9 (1C, q, $^{2}J_{C\text{-}F}$ 30.7, $CF_3$-$\underline{C}$=O), 136.6 ($\underline{C}_{ArF\mathit{F}OMe}$), 134.6 ($\underline{C}_{Ar\mathit{F}FOMe}$), 129.5 ($\underline{C}H_{ArF}$), 129.0 ($\underline{C}H_{ArF}$), 128.5 ($\underline{C}H_{ArF}$), 128.4 ($\underline{C}H_{ArF}$), 127.2 ($\underline{C}H_{ArF}$), 126.7 ($\underline{C}H_{ArF}$), 117.3 (1C, q, $^{1}J_{C\text{-}F}$ 300.6, $\underline{C}F_3$), 53.8 ($\underline{C}H_{F\mathit{F}OMe}$), 53.1 ($\underline{C}H_{\mathit{F}FOMe}$), 52.0 ($\underline{C}H_3$), 36.9 ($\underline{C}H_{2\mathit{F}FOMe}$), 36.6 ($\underline{C}H_{2F\mathit{F}OMe}$). HRMS (ESI/QTOF) *m/z*: $[M+H]^+$ calcd for $C_{19}H_{23}N_2O_3$ 327.1703 (100%), 328.1735 (22%); found 327.1704 (100%), 328.1735 (25%).

Methyl [2-(naphthalen-2-yloxy)acetyl]-*D*-phenylalanyl-*D*-phenylalaninate – **D,D-2NapFF-OMe**

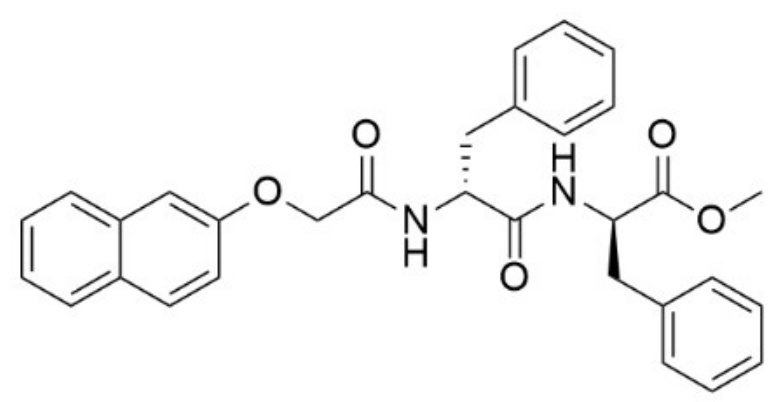

A suspension of 2-(naphthalen-2-yloxy)acetic acid (7.44 g, 36.8 mmol) in chloroform (200 mL) was stirred and cooled in an ice/brine bath for 20 min. NMM (7.0 mL, 63.7 mmol) was added and the suspension stirred until all solid was dissolved. IBCF (4.77 mL, 36.8 mmol) was added, and the solution stirred for 20 min during which a solid precipitated. A separate solution of **TFA•D,D-FF-OMe** (16.2 g, 36.8 mmol) and NMM (7.0 mL, 63.7 mmol) in chloroform (100 mL) was stirred until all solid dissolved and was added dropwise. After addition, the resulting solution was allowed to stir and warm to room temperature overnight. The now clear solution was diluted with hydrochloric acid (1M, 200 mL), stirred for 20 min, and the layers separated. The aqueous layer was extracted with chloroform (100 mL) and the combined organic extracts washed with hydrochloric acid (1M, 100 mL), water (2 × 100 mL), brine (2 × 100 mL), dried over anhydrous sodium sulfate, and filtered on a silica plug that was washed with a chloroform:ethyl acetate mixture (1:1, 200 mL). The filtrate was collected, and the solvent removed to give the crude product as a coloured solid. The solid was dissolved in chloroform (50 mL) with sonication and poured into vigorously stirred diethyl ether (500 mL), in which a solid precipitated that was left to stir overnight. The solid was collected at the filter, washed with diethyl ether (100 mL), and dried in an oven at 100 °C to give **D,D-2NapFF-OMe** as a white solid (14.7 g, 78%); mp: 153 °C. $^{1}$H NMR (400 MHz, DMSO-d$_6$) δ: 8.61 (1H, d, *J* 7.5, $N\underline{H}_{F\mathit{F}OMe}$), 8.15 (1H, d, *J* 8.5, $N\underline{H}_{\mathit{F}FOMe}$), 7.85–7.82 (2H, m, $\underline{H}_{Nap}$), 7.73 (1H, d, *J* 8.3, $\underline{H}_{Nap}$), 7.46 (1H, ddd, *J* 8.2,

6.8, 1.3, $\underline{H}_{Nap}$), 7.36 (1H, ddd, *J* 8.1, 6.8, 1.2, $\underline{H}_{Nap}$), 7.28–7.12 (12H, m, $\underline{H}_{Nap}$ and $\underline{H}_{ArF}$), 4.66 (1H, td, *J* 9.1, 4.4, $C\underline{H}_{\mathit{F}FOMe}$), 4.58–4.48 (3H, m, $C\underline{H}_{F\mathit{F}OMe}$ and O-$C\underline{H}_2$), 3.59 (3H, s, $C\underline{H}_3$), 3.08–2.91 (3H, m, 1/2$CH_{2\mathit{F}FOMe}$ and $CH_{2F\mathit{F}OMe}$), 2.85 (1H, dd, *J* 13.8, 9.5, 1/2$CH_{2\mathit{F}FOMe}$). $^{13}C$ NMR (101 MHz, DMSO-$d_6$) δ: 171.7 ($\underline{C}$=$O_{F\mathit{F}OMe}$), 170.9 ($\underline{C}$=$O_{\mathit{F}FOMe}$), 167.2 ($CH_2$-$\underline{C}$=O), 155.5 ($\underline{C}_{Nap}$-O), 137.4 ($\underline{C}_{Ar\mathit{F}FOMe}$), 136.9 ($\underline{C}_{ArF\mathit{F}OMe}$), 134.0 ($\underline{C}_{Nap}$), 129.3 ($\underline{C}H_{Nap}$), 129.2 ($\underline{C}H_{ArF}$), 129.0 ($\underline{C}H_{ArF}$), 128.7 ($\underline{C}_{Nap}$), 128.2 ($\underline{C}H_{ArF}$), 128.0 ($\underline{C}H_{ArF}$), 127.5 ($\underline{C}H_{Nap}$), 126.7 ($\underline{C}H_{Nap}$), 126.5 ($\underline{C}H_{ArF}$), 126.4 ($\underline{C}H_{Nap}$), 126.2 ($\underline{C}H_{ArF}$), 123.8 ($\underline{C}H_{Nap}$), 118.4 ($\underline{C}H_{Nap}$), 107.3 ($\underline{C}H_{Nap}$), 66.6 (O-$\underline{C}H_2$), 53.6 ($\underline{C}H_{F\mathit{F}OMe}$), 53.1 ($\underline{C}H_{\mathit{F}FOMe}$), 51.8 ($\underline{C}H_3$), 37.4 ($\underline{C}H_{2F\mathit{F}OMe}$), 36.6 ($\underline{C}H_{2\mathit{F}FOMe}$). HRMS (ESI/QTOF) *m/z*: $[M+H]^+$ calcd for $C_{31}H_{31}N_2O_5$ 511.2227 (100%), 512.2260 (35%); found 511.2227 (100%), 512.2256 (36%).

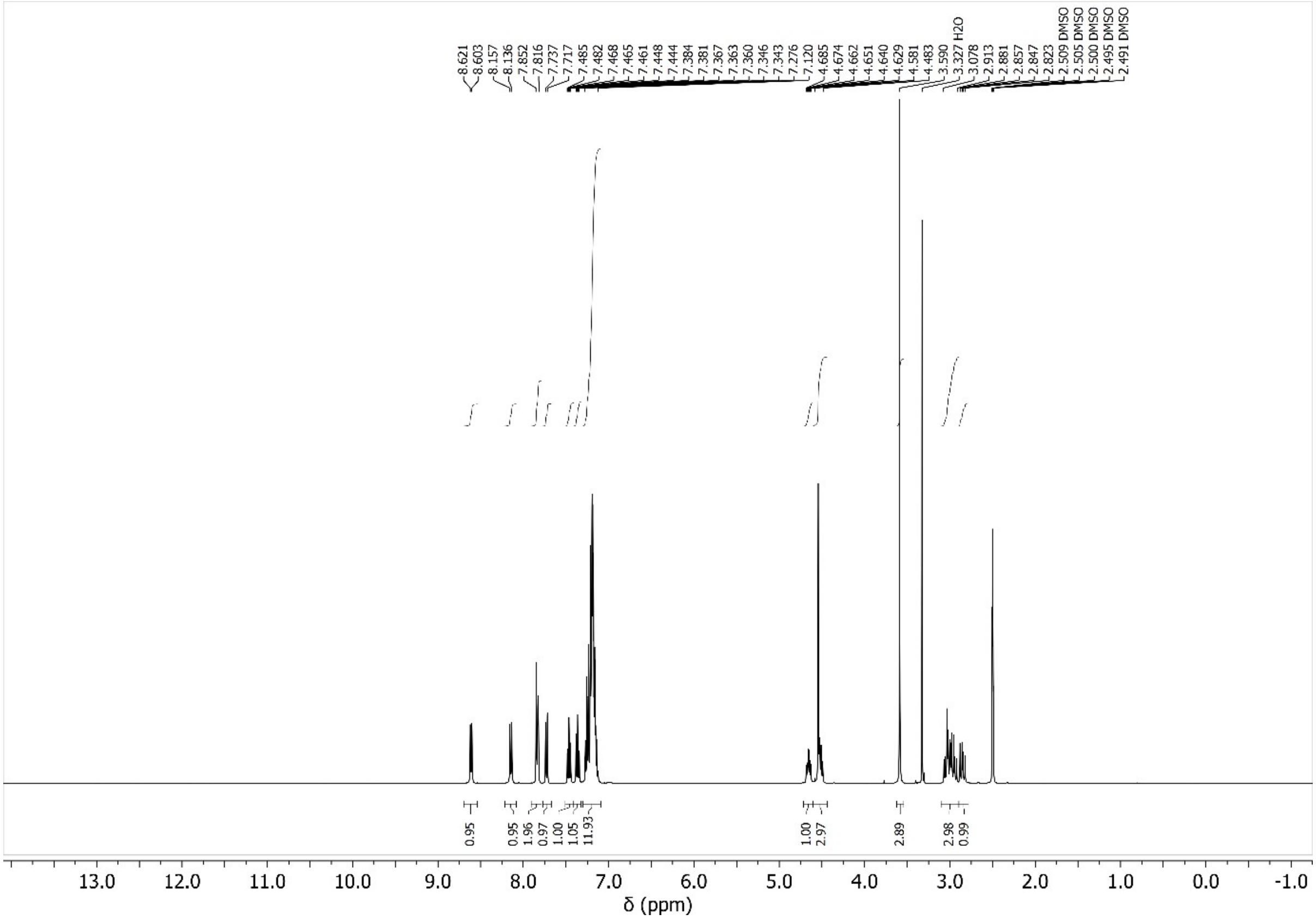


**Figure S15**. $^1H$ NMR spectrum (DMSO-$d_6$, 400 MHz) of **D,D-2NapFF-OMe**.

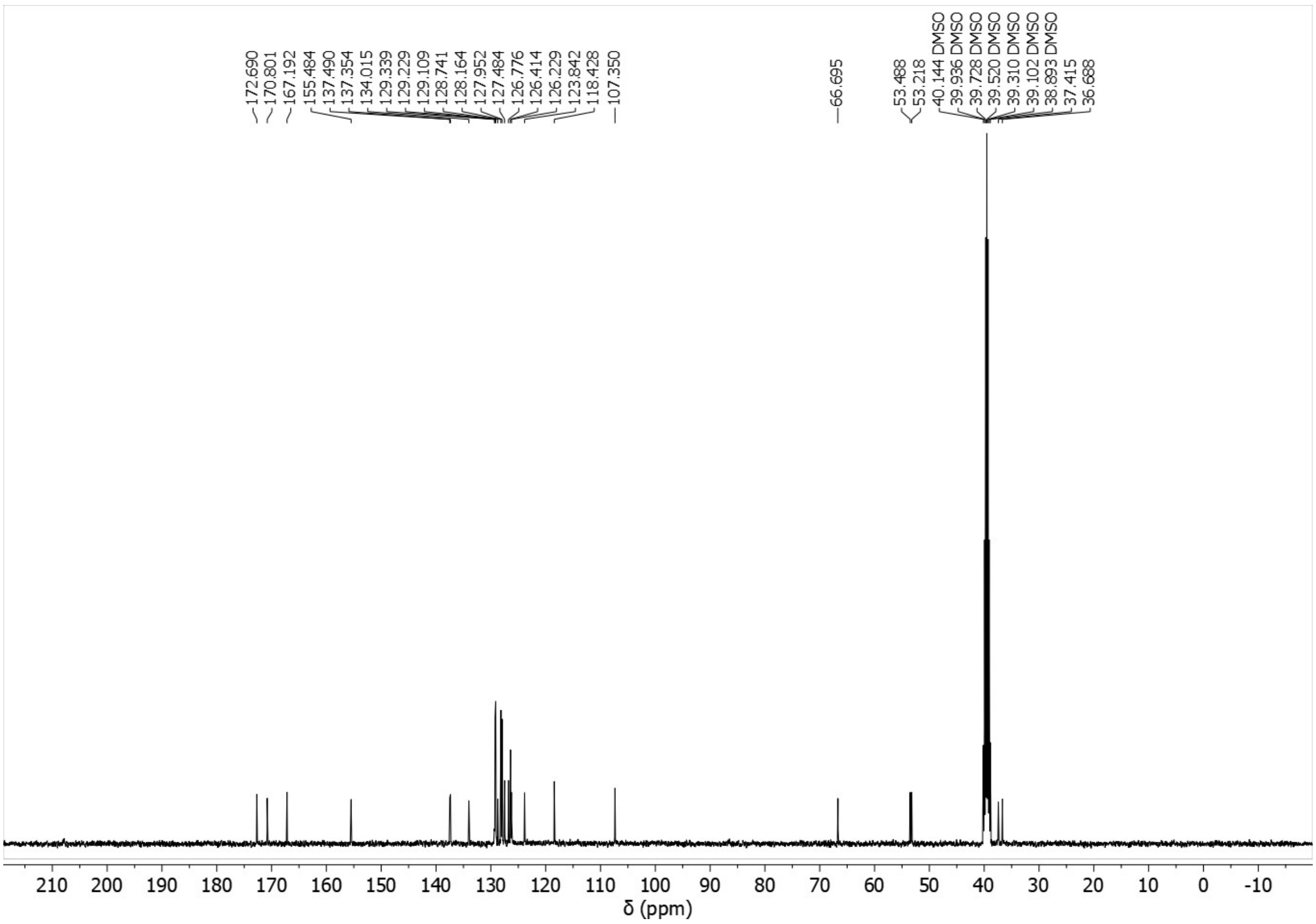


**Figure S16**. $^{13}$C NMR spectrum (DMSO-$d_6$, 101 MHz) of **D,D-2NapFF-OMe**.

[2-(Naphthalen-2-yloxy)acetyl]-*D*-phenylalanyl-*D*-phenylalanine – **D,D-2NapFF**



A suspension of **D,D-2NapFF-OMe** (8.60 g, 16.8 mmol) and lithium hydroxide (1.0 g, 41.8 mmol) in tetrahydrofuran (75 mL) and water (75 mL) was stirred for 2 d. The now clear solution was filtered through a 0.2 µm nylon filter and the filtrate slowly poured into vigorously stirred hydrochloric acid (1M, 500 mL) to give a white precipitate. The suspension was allowed to stir for 1 h, and the solid was collected at the filter and washed with water (2 × 200 mL). The crude solid was sonicated in acetonitrile (100 mL) for 1 h, collected at the filter, and dried in an oven at 70 °C to give **D,D-2NapFF** as a white solid (5.95 g, 72%); mp: 166 °C. $^{1}$H NMR (400 MHz, DMSO-$d_6$) δ: 12.81 (1H, s, COO$\underline{H}$), 8.45 (1H, d, *J* 7.8, N$\underline{H}_{F\mathit{F}OH}$), 8.12 (1H, d, *J* 8.6, N$\underline{H}_{\mathit{F}FOH}$), 7.86–7.81 (2H, m, $\underline{H}_{Nap}$), 7.73 (1H, d, *J* 8.2, $\underline{H}_{Nap}$), 7.46 (1H, ddd, *J* 8.2, 6.8, 1.3, $\underline{H}_{Nap}$), 7.36 (1H, ddd, *J* 8.1, 6.8, 1.3, $\underline{H}_{Nap}$), 7.28–7.10 (12H, m, $\underline{H}_{Nap}$ and $\underline{H}_{ArF}$), 4.65 (1H, td, *J* 9.1, 4.2, C$\underline{H}_{\mathit{F}FOH}$), 4.54 (2H, s, O-C$\underline{H}_2$), 4.48 (1H, td, *J* 8.3, 5.3, C$\underline{H}_{F\mathit{F}OH}$), 3.11–3.00 (2H, m, 1/2C$\underline{H}_{2F}$), 2.96–2.81 (2H, m, 1/2C$\underline{H}_{2F}$). $^{13}$C NMR (101 MHz, DMSO-$d_6$) δ: 172.7 ($\underline{C}$OOH), 170.8 ($\underline{C}$=$O_{\mathit{F}FOH}$), 167.2 ($CH_2$-$\underline{C}$=O), 155.5 ($\underline{C}_{Nap}$-O), 137.5 ($\underline{C}_{Ar\mathit{F}FOH}$), 137.3 ($\underline{C}_{ArF\mathit{F}OH}$), 134.0 ($\underline{C}_{Nap}$), 129.3 ($\underline{C}H_{Nap}$), 129.2 ($\underline{C}H_{ArF}$), 129.1 ($\underline{C}H_{ArF}$), 128.7 ($\underline{C}_{Nap}$), 128.2 ($\underline{C}H_{ArF}$), 128.0 ($\underline{C}H_{ArF}$), 127.5 ($\underline{C}H_{Nap}$), 126.8 ($\underline{C}H_{Nap}$), 126.43 ($\underline{C}H_{Nap}$), 126.41 ($\underline{C}H_{ArF}$), 126.2 ($\underline{C}H_{ArF}$), 123.8 ($\underline{C}H_{Nap}$), 118.4 ($\underline{C}H_{Nap}$), 107.3 ($\underline{C}H_{Nap}$), 66.7 (O-$\underline{C}H_2$), 53.5 ($\underline{C}H_{F\mathit{F}OH}$), 53.2 ($\underline{C}H_{\mathit{F}FOH}$), 37.4 ($\underline{C}H_{2\mathit{F}FOH}$), 36.7 ($\underline{C}H_{2F\mathit{F}OH}$). HRMS (ESI/QTOF) *m/z*: $[M+H]^+$ calcd for $C_{30}H_{29}N_2O_5$ 497.2071 (100%), 498.2104 (34%); found 497.2068 (100%), 498.2104 (35%).

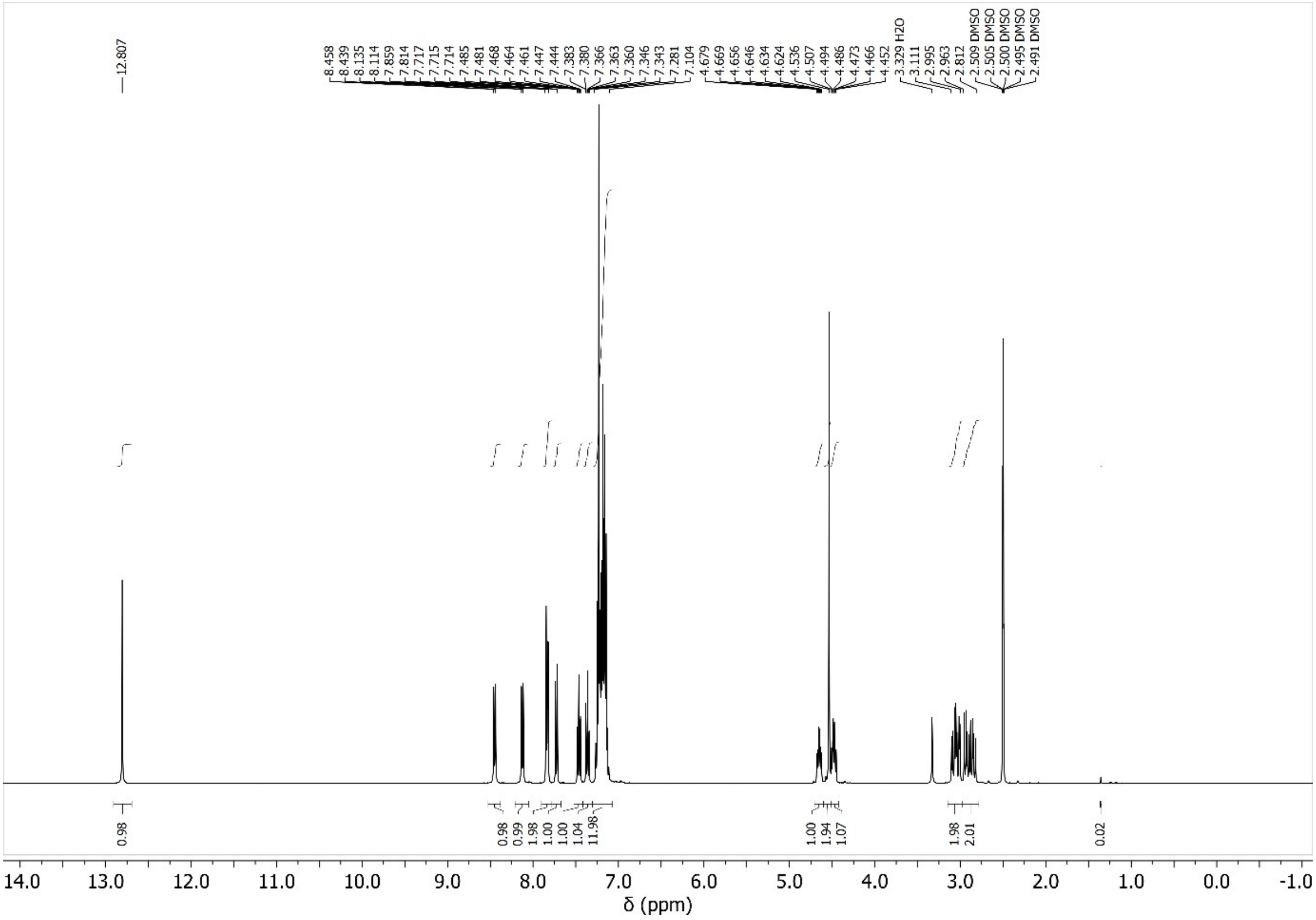

**Figure S17**. $^{1}$H NMR spectrum (DMSO-$d_6$, 400 MHz) of **D,D-2NapFF**.

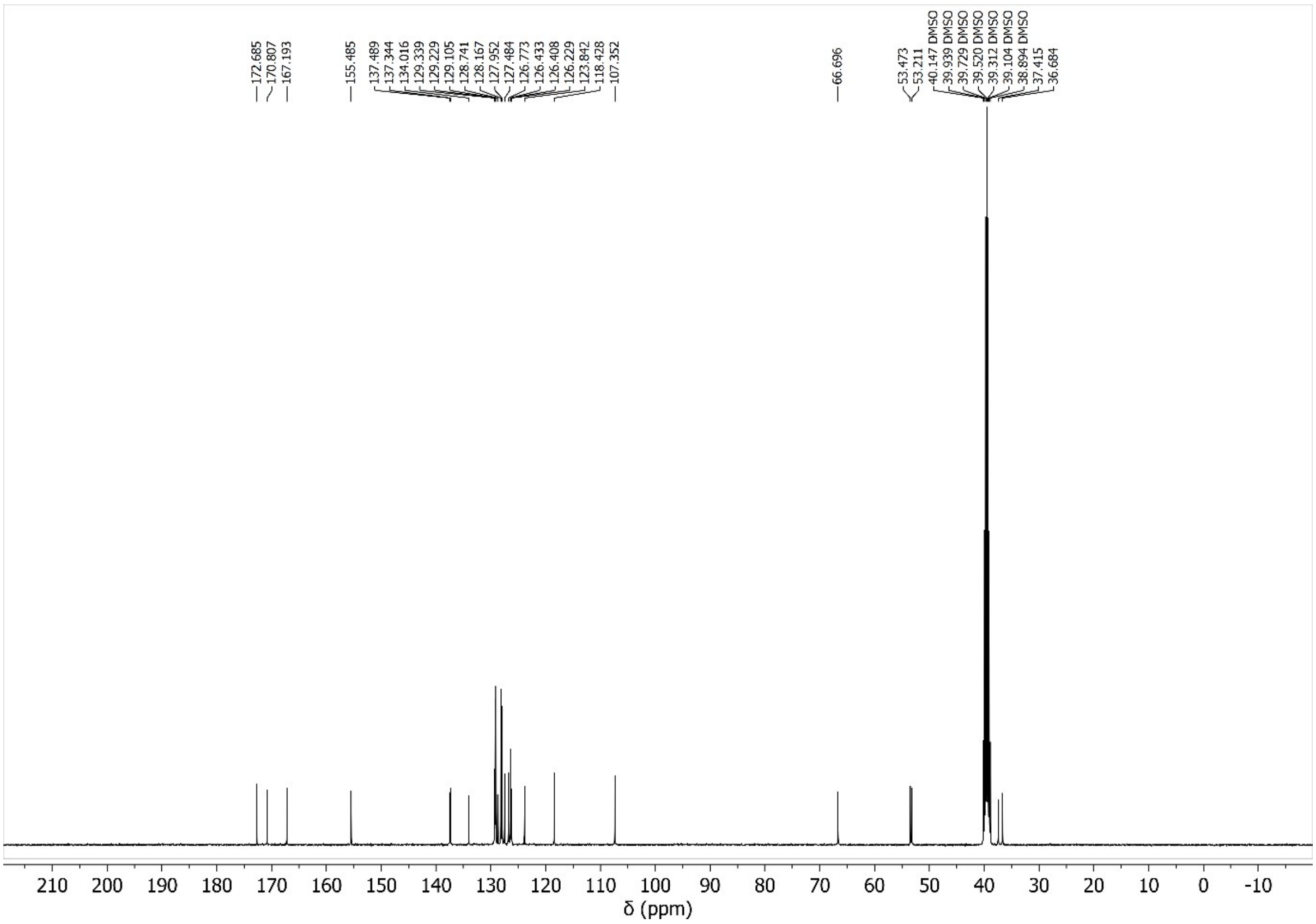

**Figure S18**. $^{13}$C NMR spectrum (DMSO-$d_6$, 101 MHz) of **D,D-2NapFF**.

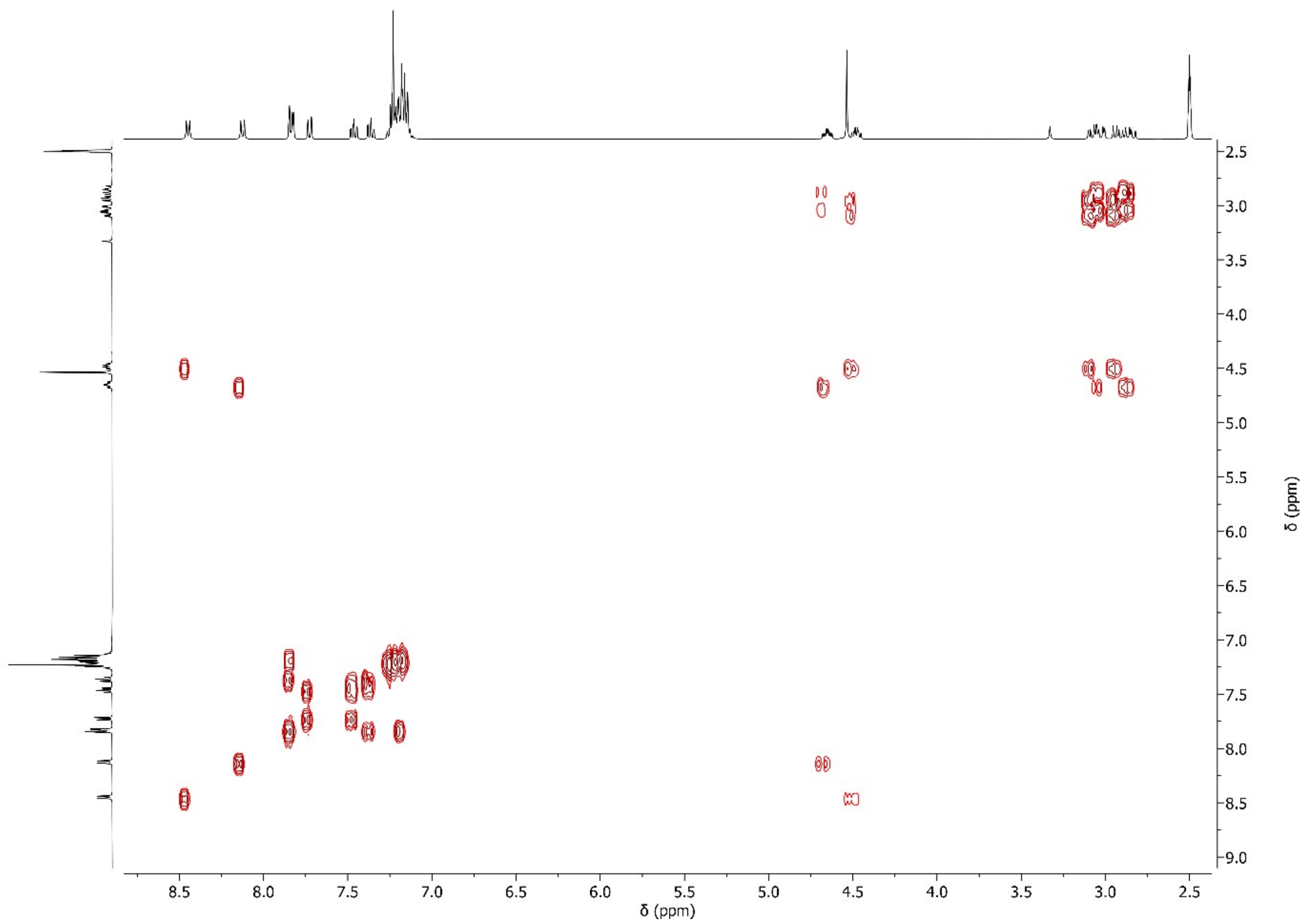


**Figure S19**. COSY NMR (DMSO-$d_6$) of **D,D-2NapFF**.

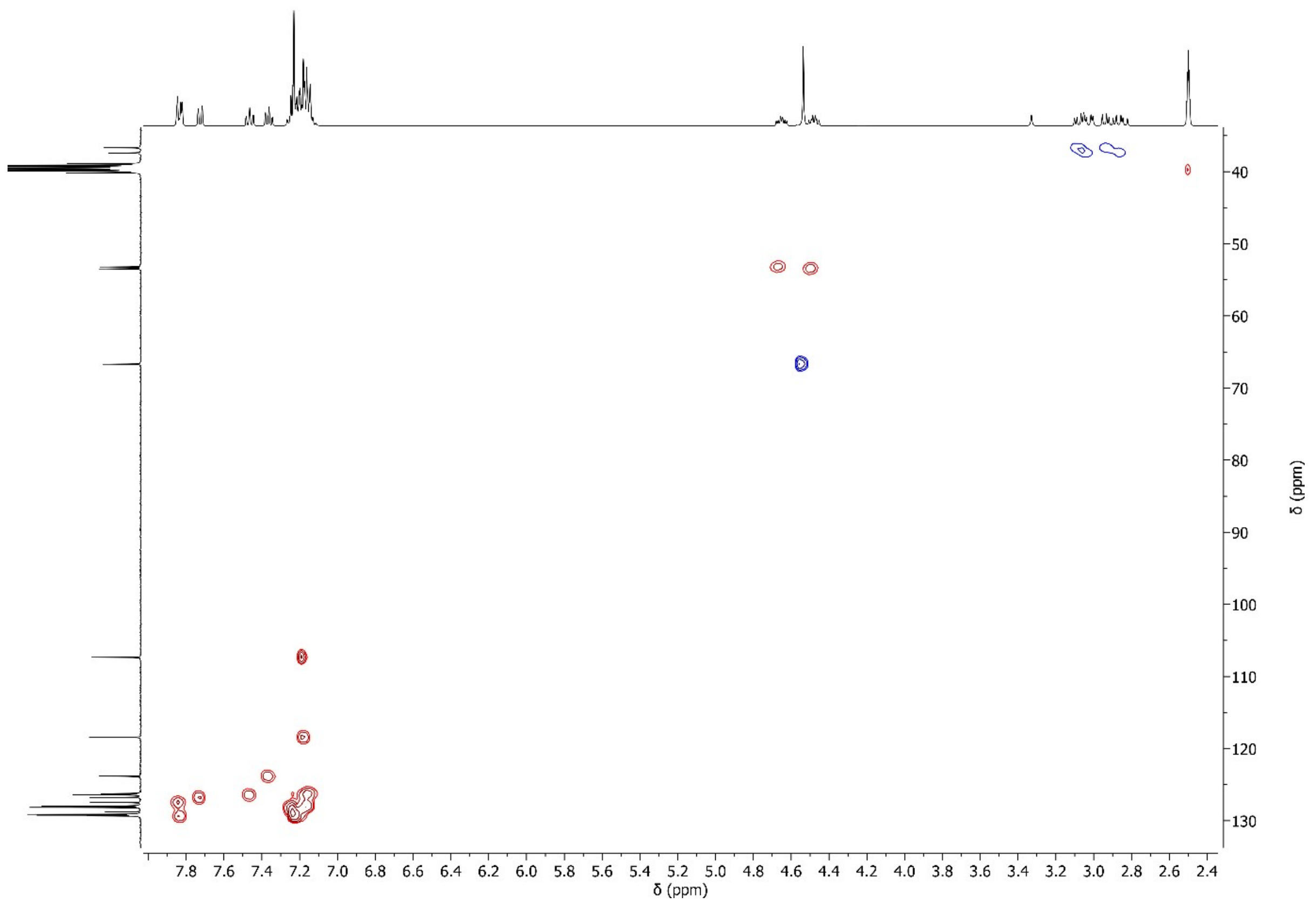


**Figure S20**. HSQC NMR (DMSO-$d_6$) of **D,D-2NapFF**.

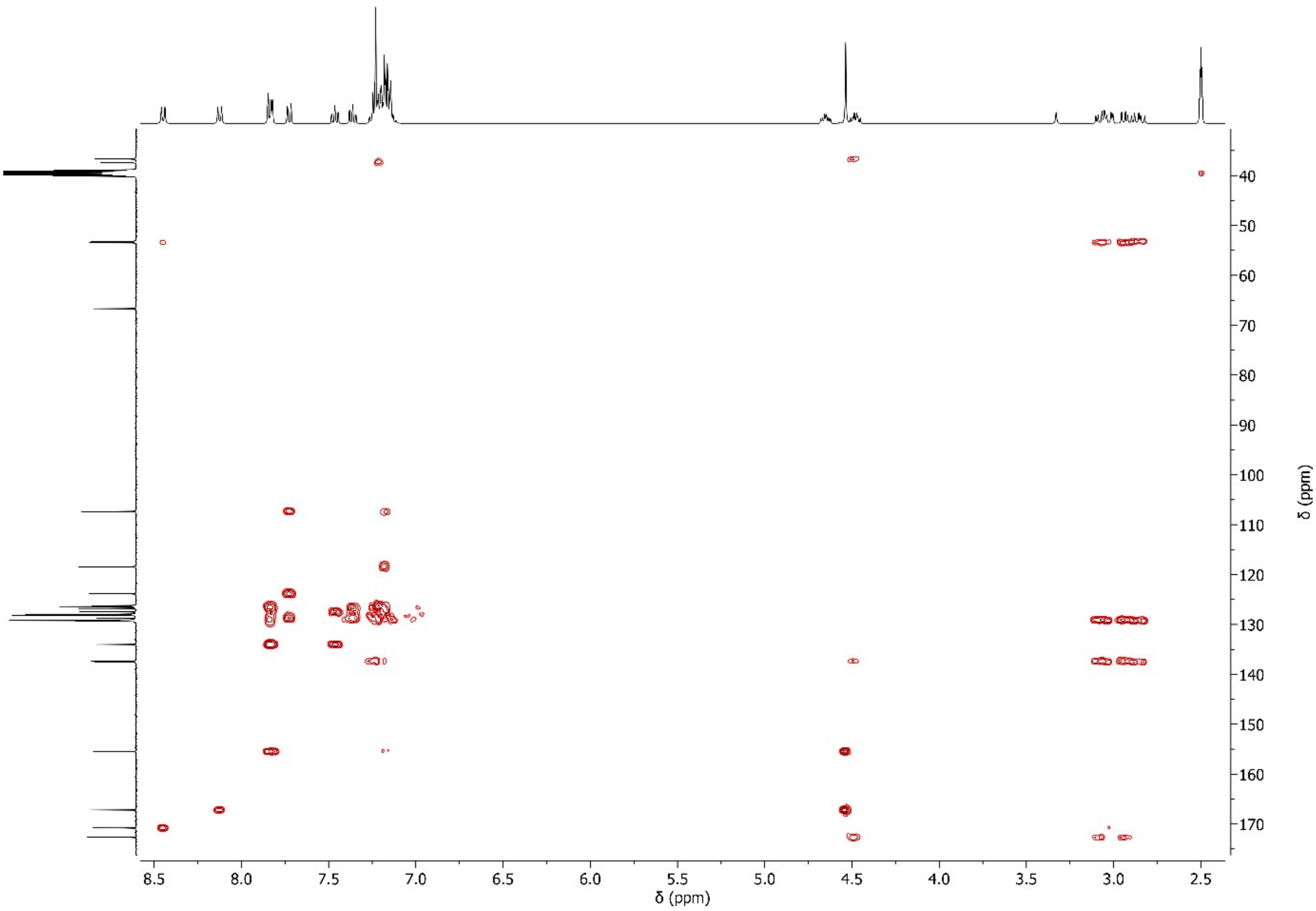


**Figure S21**. HMBC NMR (DMSO-$d_6$) of **D,D-2NapFF**.

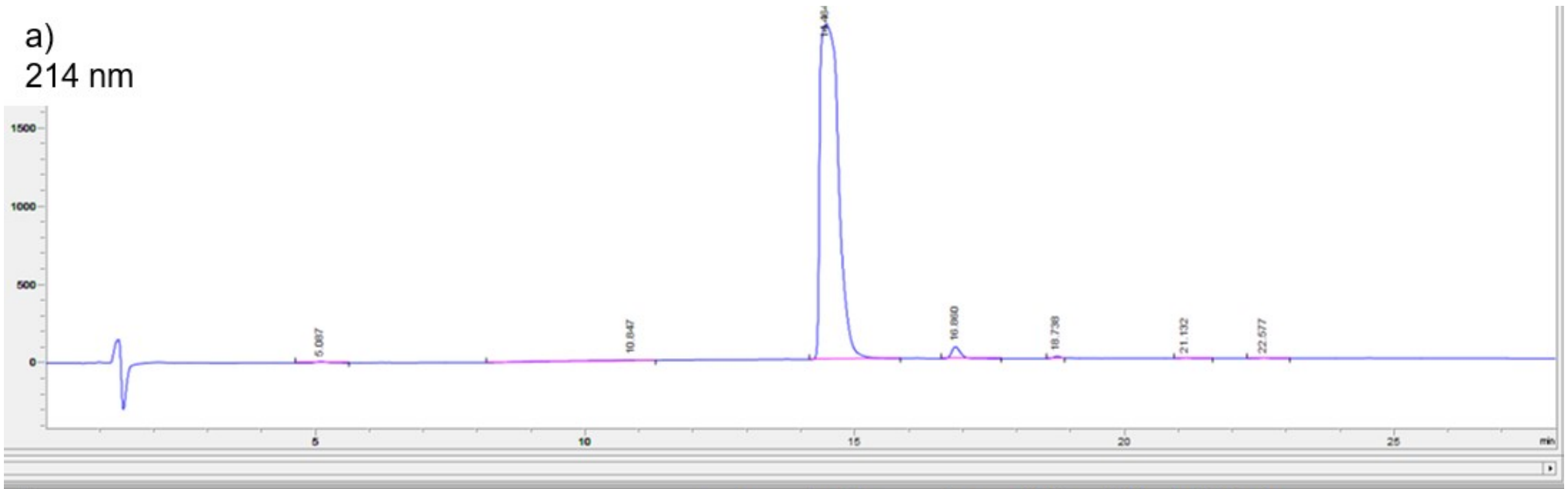


| # | Time | Type | Area | Height | Width | Area% | Symmetry |
|---|---|---|---|---|---|---|---|
| 1 | 5.087 | BB | 122.3 | 9 | 0.2128 | 0.234 | 0.768 |
| 2 | 10.847 | BB | 488.8 | 4.2 | 1.3933 | 0.934 | 6.98 |
| 3 | 14.464 | BB | 50699.5 | 2140.2 | 0.3244 | 96.849 | 0.456 |
| 4 | 16.86 | BB | 863.7 | 75.9 | 0.1727 | 1.650 | 0.713 |
| 5 | 18.738 | BB | 110.4 | 13.3 | 0.1306 | 0.211 | 0.993 |
| 6 | 21.132 | BB | 31.9 | 2.9 | 0.1687 | 0.061 | 0.744 |
| 7 | 22.577 | BB | 32.4 | 3 | 0.1639 | 0.062 | 0.741 |

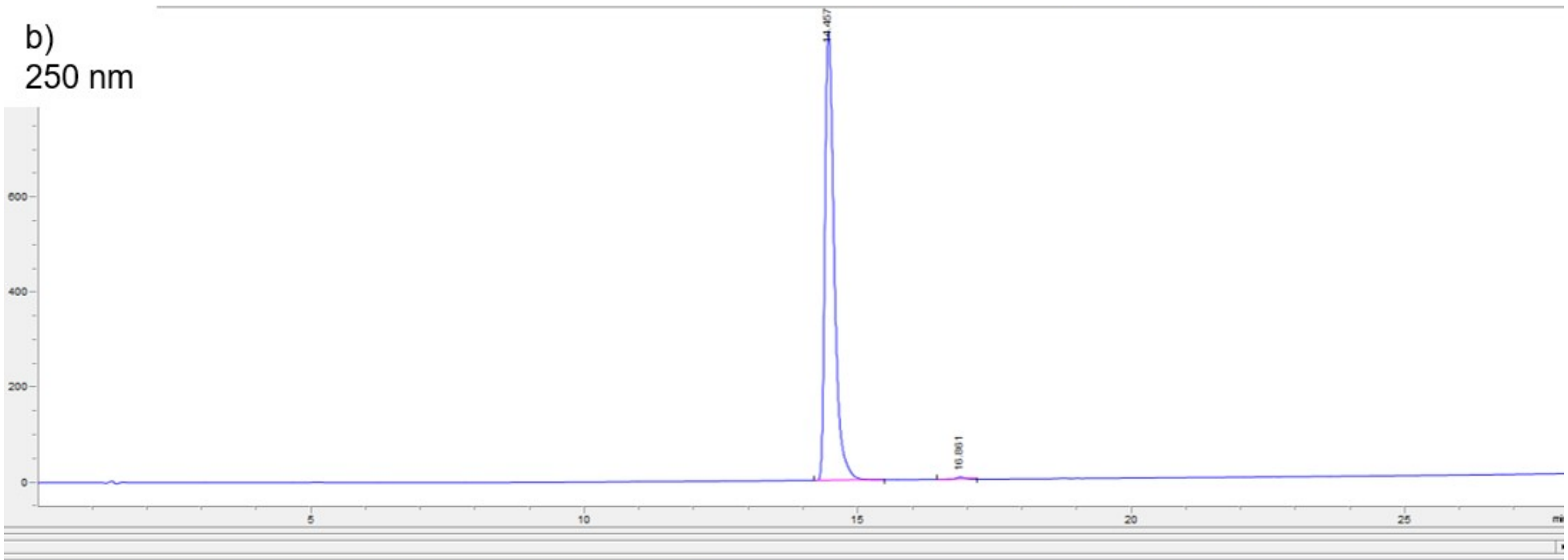


| # | Time | Type | Area | Height | Width | Area% | Symmetry |
|---|---|---|---|---|---|---|---|
| 1 | 14.457 | BB | 11214.4 | 943.7 | 0.1826 | 99.534 | 0.581 |
| 2 | 16.861 | BB | 52.6 | 4.7 | 0.1642 | 0.466 | 0.783 |

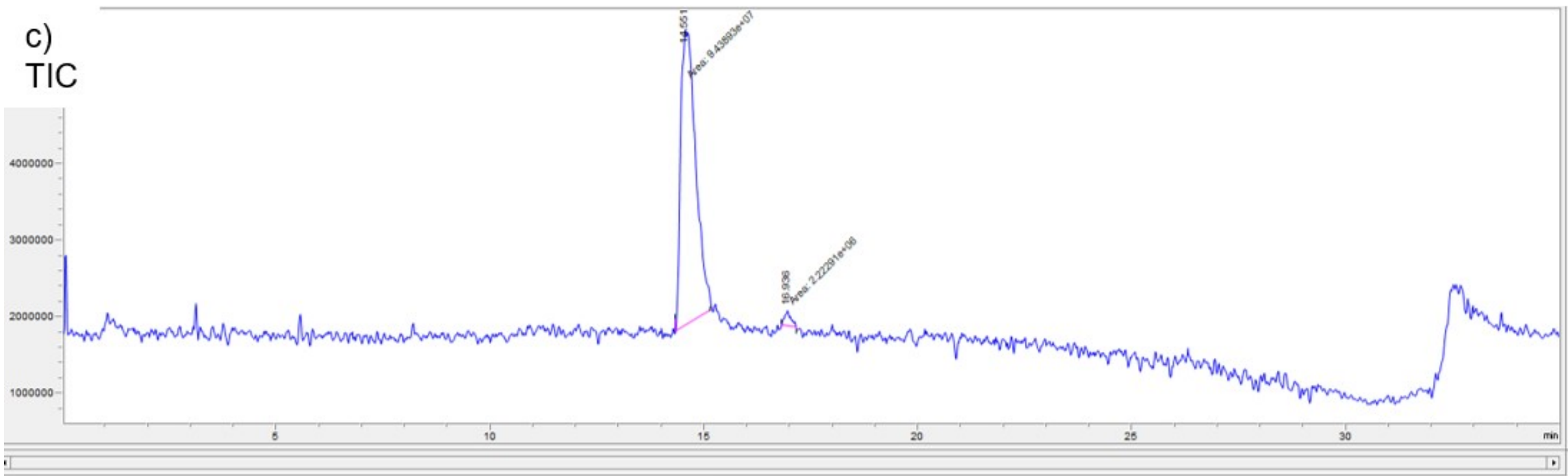


| # | Time | Type | Area | Height | Width | Area% | Symmetry |
|---|---|---|---|---|---|---|---|
| 1 | 14.551 | MM | 94389264 | 3896189.8 | 0.4038 | 97.699 | 0.448 |
| 2 | 16.936 | MM | 2222905 | 214085.5 | 0.1731 | 2.301 | 0.778 |

**Figure S22**. HPLC-ESI-MS chromatograms of **D,D-2NapFF** from the a) 214 nm and b) 250 nm detector, and c) total ion chromatogram (TIC). Conditions: C18 reversed-phase column; linear gradient from 40% to 90% acetonitrile in water over 20 min; flow rate 0.30 mL $min^{-1}$; injection volume 5 µL; sample concentration 2 mg $mL^{-1}$; room temperature.

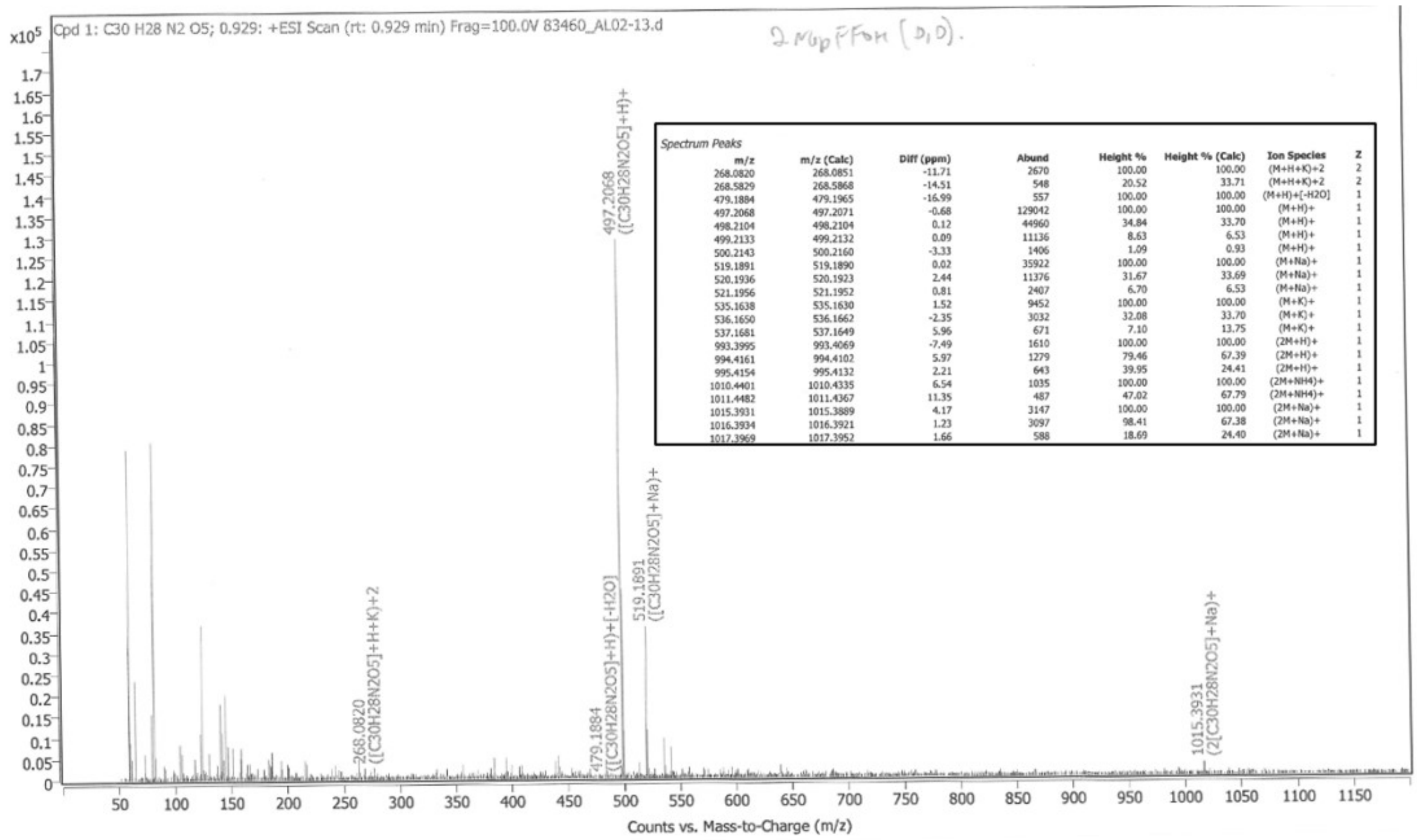


| m/z | m/z (Calc) | Diff (ppm) | Abund | Height % | Height % (Calc) | Ion Species | Z |
|---|---|---|---|---|---|---|---|
| 268.0820 | 268.0851 | -11.71 | 2670 | 100.00 | 100.00 | (M+H+K)+2 | 2 |
| 268.5829 | 268.5868 | -14.51 | 548 | 20.52 | 33.71 | (M+H+K)+2 | 2 |
| 479.1884 | 479.1965 | -16.99 | 557 | 100.00 | 100.00 | (M+H)+[-H2O] | 1 |
| 497.2068 | 497.2071 | -0.68 | 129042 | 100.00 | 100.00 | (M+H)+ | 1 |
| 498.2104 | 498.2104 | 0.12 | 44960 | 34.84 | 33.70 | (M+H)+ | 1 |
| 499.2133 | 499.2132 | 0.09 | 11136 | 8.63 | 6.53 | (M+H)+ | 1 |
| 500.2143 | 500.2160 | -3.33 | 1406 | 1.09 | 0.93 | (M+H)+ | 1 |
| 519.1891 | 519.1890 | 0.02 | 35922 | 100.00 | 100.00 | (M+Na)+ | 1 |
| 520.1936 | 520.1923 | 2.44 | 11376 | 31.67 | 33.69 | (M+Na)+ | 1 |
| 521.1956 | 521.1952 | 0.81 | 2407 | 6.70 | 6.53 | (M+Na)+ | 1 |
| 535.1638 | 535.1630 | 1.52 | 9452 | 100.00 | 100.00 | (M+K)+ | 1 |
| 536.1650 | 536.1662 | -2.35 | 3032 | 32.08 | 33.70 | (M+K)+ | 1 |
| 537.1681 | 537.1649 | 5.96 | 671 | 7.10 | 13.75 | (M+K)+ | 1 |
| 993.3995 | 993.4069 | -7.49 | 1610 | 100.00 | 100.00 | (2M+H)+ | 1 |
| 994.4161 | 994.4102 | 5.97 | 1279 | 79.46 | 67.39 | (2M+H)+ | 1 |
| 995.4154 | 995.4132 | 2.21 | 643 | 39.95 | 24.41 | (2M+H)+ | 1 |
| 1010.4401 | 1010.4335 | 6.54 | 1035 | 100.00 | 100.00 | (2M+NH4)+ | 1 |
| 1011.4482 | 1011.4367 | 11.35 | 487 | 47.02 | 67.79 | (2M+NH4)+ | 1 |
| 1015.3931 | 1015.3889 | 4.17 | 3147 | 100.00 | 100.00 | (2M+Na)+ | 1 |
| 1016.3934 | 1016.3921 | 1.23 | 3097 | 98.41 | 67.38 | (2M+Na)+ | 1 |
| 1017.3969 | 1017.3952 | 1.66 | 588 | 18.69 | 24.40 | (2M+Na)+ | 1 |

**Figure S23**. ESI-HRMS spectrum of **D,D-2NapFF** with peak table inset.
